# Tetraarsenic hexoxide enhances generation of mitochondrial ROS to promote pyroptosis by inducing the activation of caspase-3/GSDME in triple-negative breast cancer cells

**DOI:** 10.1038/s41419-021-03454-9

**Published:** 2021-02-08

**Authors:** Haein An, Jin Sun Heo, Pyunggang Kim, Zenglin Lian, Siyoung Lee, Jinah Park, Eunji Hong, Kyoungwha Pang, Yuna Park, Akira Ooshima, Jihee Lee, Minjung Son, Hyeyeon Park, Zhaoyan Wu, Kyung-Soon Park, Seong-Jin Kim, Illju Bae, Kyung-Min Yang

**Affiliations:** 1 Precision Medicine Research Center, Advanced Institute of Convergence Technology, Seoul National University, Suwon, Gyeonggi-do 16229 Republic of Korea; 2 Department of Biological Science, Sungkyunkwan University, Suwon,, 16419 Gyeonggi-do Republic of Korea; 3 Department of Biomedical Science, College of Life Science, CHA University, Seongnam City, 463-400 Gyeonggi-do Republic of Korea; 4 Beijing Yichuang Biotechnology Industry Research Institute, Beijing, China; 5 Chemas Co., Ltd., Seoul, Republic of Korea; 6 Department of Transdisciplinary Studies, Graduate School of Convergence Science and Technology, Suwon, Gyeonggi-do 16229 Republic of Korea; 7 Medpacto Inc., Seoul, Republic of Korea

**Keywords:** Breast cancer, Apoptosis

## Abstract

Although tetraarsenic hexoxide is known to exert an anti-tumor effect by inducing apoptosis in various cancer cells, its effect on other forms of regulated cell death remains unclear. Here, we show that tetraarsenic hexoxide induces the pyroptotic cell death through activation of mitochondrial reactive oxygen species (ROS)-mediated caspase-3/gasdermin E (GSDME) pathway, thereby suppressing tumor growth and metastasis of triple-negative breast cancer (TNBC) cells. Interestingly, tetraarsenic hexoxide-treated TNBC cells exhibited specific pyroptotic characteristics, including cell swelling, balloon-like bubbling, and LDH releases through pore formation in the plasma membrane, eventually suppressing tumor formation and lung metastasis of TNBC cells. Mechanistically, tetraarsenic hexoxide markedly enhanced the production of mitochondrial ROS by inhibiting phosphorylation of mitochondrial STAT3, subsequently inducing caspase-3-dependent cleavage of GSDME, which consequently promoted pyroptotic cell death in TNBC cells. Collectively, our findings highlight tetraarsenic hexoxide-induced pyroptosis as a new therapeutic strategy that may inhibit cancer progression of TNBC cells.

## Introduction

Triple-negative breast cancer (TNBC) accounts for 12 to 17% of patients with breast cancer worldwide, and frequently occurs in young African American women as well as women with *BRCA1* mutations^1^. Among the breast cancer subtypes, TNBC is highly heterogeneous and aggressive, resulting in the worst prognosis due to the lack of specific targets compared to hormone receptors- and HER2-enriched subtypes^1,2^. Although several clinical trials are conducted by using therapeutic agents against specific molecular targets in TNBC, such as immune checkpoint inhibitors and poly ADP-ribose polymerase (PARP) inhibitors, conventional chemotherapy drugs are still mainly used as the primary treatment for patients with TNBC due to their little effect^3–5^. Nevertheless, insensitivity of TNBC to chemotherapy is often associated with increased risk of recurrence and metastasis, resulting in high mortality rates for patients with TNBC^6^. Therefore, there is an urgent need to develop effective neoadjuvant chemotherapy agents against TNBC that can improve a very poor prognosis for patients with TNBC.

Arsenic derivative compounds have been shown to exert anti-cancer effects. For example, arsenic trioxide (As_2_O_3_, Trisenox^®^) has been used as standard monotherapy in acute promyelocytic leukemia (APL), which is a rare case of acute myeloid leukemia (AML), targeting the PML/RARA oncogene^7,8^. In addition, studies have shown that modified arsenic derivative compounds such as arsenic trisulfide (As_2_S_3_) and tetraarsenic hexoxide (As_4_O_6_, TetraAS^®^) demonstrated potent anti-cytotoxic effect in various cancer cells, including leukemia, glioma, colon, breast, and cervix cancer cells^9–13^. Particularly, tetraarsenic hexoxide was developed as a chemotherapeutic agent for clinical trials for patients with advanced cancers. Studies have demonstrated that tetraarsenic hexoxide induces apoptosis by activating reactive oxidative species (ROS) and proapoptotic proteins, such as caspase-3 and caspase-8, and autophagic cell death^14^. Furthermore, it is reported that the inhibitory effect of tetraarsenic hexoxide on cell growth is more potent than that of arsenic trioxide in cervical cancer cells^15^. Although anti-cancer effect of tetraarsenic hexoxide has been extensively studied in various cancer cells, the molecular basis of its tumor inhibitory activity remains poorly understood.

Pyroptosis, a type of inflammasomes-induced programmed necrosis, critically depends on pore formation of the plasma membrane by activating gasdermin proteins, especially gasdermin D (GSDMD) as the pyroptotic substrate of inflammatory caspase-1/4/5/11^16–18^. Although pyroptosis has been widely studied in cell death-associated inflammatory responses, which is different from apoptosis, there is increasing number of studies researching on the role of pyroptosis in inhibiting the proliferation of cancer cells. Intriguingly, it has been recently reported that activation of caspase-3, a critical executioner of apoptosis, by TNF-α or chemotherapy drugs facilitates the cleavage of gasdermin E (GSDME, encoded by *DFNA5*), consequently switching apoptosis to pyroptosis as a secondary necrosis^19,20^. Furthermore, it was shown that the loss of GSDME, a candidate tumor suppressor that is induced by etoposide-activated p53, caused resistance to etoposide in melanoma cells^21–23^. However, despite these findings, the role of pyroptosis in the context of metastatic breast cancers has not been extensively investigated yet.

In this study, we show that tetraarsenic hexoxide induces GSDME-mediated pyroptosis by increasing generation of mitochondrial ROS through specific inhibition of mitochondrial STAT3 phosphorylation, thereby suppressing proliferation and metastasis of aggressive TNBC cells.

## Materials and methods

### Cell culture and reagents

Mouse mammary carcinoma cells (EO771, 4T1), human breast cancer cells (Hs578T, MDA-MB-231), mouse normal mammary epithelial cells (NMuMG), and human non-malignant breast epithelial cells (MCF10A) were purchased from American Type Culture Collection (ATCC). EO771, 4T1, Hs578T, and MDA-MB-231 breast cancer cells were maintained in monolayers in DMEM (WelGENE, Daegu, South Korea) with 10% fetal bovine serum (FBS; WelGENE, Daegu, South Korea), 1% penicillin/streptomycin (GIBCO, Grand Island, NY, USA). NMuMG cells were maintained in monolayers in DMEM (WelGENE) with 10% fetal bovine serum (FBS; WelGENE, Daegu, South Korea), 1% penicillin/streptomycin, and 10 μg/ml insulin (Sigma-Aldrich, St.Louis, MO, USA). MCF10A cells were maintained in DMEM/Ham’s F-12 nutrient mixture (GIBCO) with 5% horse serum (GIBCO), 20 ng/ml EGF (Peprotech), 10 μg/ml insulin, 0.5 μg/ml hydrocortisone (Sigma), and 100 ng/ml choleratoxin (Sigma). All cell lines were maintained at 37 °C in CO_2_ humidified atmosphere. For all experiments cells were grown to 70–80% confluence. The cell lines in this study were routinely tested for mycoplasma contamination by PCR. Tetraarsenic hexoxide (As_4_O_6_, TetraAS^®^) was provided by CHEMAS Co. Ltd. (Seoul, South Korea) N-Acetyl-Asp-Glu-Val-Asp-al (Ac-DEVD-CHO) and N-Acetyl-L-cysteine (NAC) were purchased from Sigma-Aldrich.

### In vivo tumor formation and lung metastasis

4T1-Luc cells (1 × 10^5^) were orthotopically injected into the mammary fad of 6-weeks old female Balb/c mice. Four weeks after inoculation, mice were treated daily with or without 2 and 4 mg/kg tetraarsenic hexoxide in a dose-dependent manner by oral gavage. Tumor volumes were calculated using the formula Volume (cm^3^) = (S × S × L) × 0.5, where *S* and *L* are the short and long dimensions of the tumor, respectively. Monitoring of the occurrence of spontaneous lung metastasis was performed by bioluminescence imaging after intraperitoneal injection of D-luciferin (Promega, Madison, WI, USA). The intensities of bioluminescence signals were measured using an IVS-200 system (Xenogen Corp., San Francisco, CA, USA). After the experiment, mice were sacrificed via CO_2_ asphyxiation followed by cervical dislocation. Then, lungs were perfused with 7.5% of India ink and destained in Fekete’s solution. Metastatic lesions were counted and presented by the mean number of lesions in each group. All of the animals were maintained according to the Woo Jung Bio Facility (Suwon, South Korea) and Use Committee guidelines under protocol number IACUC110004.

### Transmission electron microscopy (TEM)

4T1 cells were seeded at the density of 1 × 10^5^ cells/well in a 100 mm dish for 24 h and cells were fixed by incubating with fixation solution including 4% formaldehyde, 2% glutaraldehyde, 0.1 M cacodylate for 2 h at room temperature. 1% osmium tetroxide was used as a postfixed solution for 1 h and stained in 0.5% uranyl acetate for 4 h. Samples were gradually dehydrated in ethanol (from 35 to 100%) and finally replaced to 100% propylene oxide. After infiltration in propylene oxide and epoxy resin for overnight, samples were precipitated in 100% epoxy resin. Polymerization of resin was conducted for 72 h at 60 °C. Thin sections of 80–100 nm were cut using an ultramicrotome (RMC), stained with lead citrate, uranyl acetate, lightly carbon coated, and imaged in a TalosL120C transmission electron microscope (FEI).

### Cell viability assay

Cell viability assay was performed using the 3-[4,5-dimethylthiazol-2-yl]-2,5-diphenyltetrazolium bromide (MTT) (Sigma-Aldrich, St. Louis, MO, USA) and lactate dehydrogenase (LDH) (Roche, Mannheim, Germany). Cells were pretreated with or without NAC or DEVD for 2 h, then, tetraarsenic hexoxide was treated in a dose-dependent manner. For MTT assay, 0.5 mg/ml of MTT was treated to each well and incubated for 2 h at 37 °C. After incubation, the culture medium was discarded and dimethyl sulfoxide (DMSO) was added to dissolve the formazan crystals. Plates were analyzed at the absorption values at 570 nm using a 96-well microplate reader. For LDH assay, LDH reagent was incubated with cell supernatants for 30 min at room temperature in the dark and analyzed the absorption values at 492 nm using a 96-well microplate reader. The cell viability was also analyzed by using a propidium iodide (PI) fluorescent probe. Briefly, cells were grown in 6-well glass-bottom plates at a density of 2 × 10^4^/well and incubated with or without tetraarsenic hexoxide treatment. Then cells were stained using 10 μg/ml PI (Komabiotech, Seoul, South Korea). The level of fluorescence intensity of PI was detected using CELENA^®^S Digital Imaging System (Logos Biosystems, Gyeonggi-do, South Korea).

### Foci formation assay and cell migration assay

For foci formation, cells were seeded to 4 × 10^2^ in 6-well plates and incubated for 5–6 days with or without tetraarsenic hexoxide treatment. The colonies were washed with PBS and stained with 2% methylene blue in 50% ethanol. All of the experiments were conducted in triplicate. For Transwell migration assays were performed using Transparent PET membrane inserts (Falcon, #353097) as described in the manufacturer’s protocol. A total of 5 × 10^4^ cells were plated in the transwell and incubated for 16 h. The cells that penetrated and migrated to the opposite surface of the transwell were fixed with 70% ethanol and stained with 0.05% methylene blue. The numbers of invaded cells in each field of view were quantified for statistics analysis.

### Immunoblot analysis

The lysates were prepared using IP buffer (50 mM Tris, pH 7.4, 150 mM NaCl, 1% Triton X-100, 2 mM EDTA, and 10% glycerol) plus phosphatase and protease inhibitors (Roche, Mannheim, Germany). For mitochondrial fractionation, mitochondria were isolated from cytosolic components of the cells using a mitochondria isolation kit (Thermo Fisher, Rockford, IL, USA) according to the manufacturer’s protocols. Protein concentrations were determined by BCA assay and then lysates were subjected to SDS-PAGE, transferred onto 0.45 μm PVDF membrane (GE healthcare, Buckinghamshire, UK). The membranes were incubated with the appropriate primary antibodies for overnight. The primary antibodies used are as following: Caspase-3 (Rb, 1:1000; Cell Signaling, #9662), Cleaved Caspase-3 (Rb, 1:1000; Cell Signaling, #9664), Cytochrome C (Rb, 1:1000; Cell Signaling, #11940), Gasdermin D (Rb, 1:1000; Cell Signaling, #93709), PARP (Rb, 1:1000; Cell Signaling, #9542), p-STAT3 (Thy705) (Rb, 1:1000; Cell Signaling, #9145), DFNA5/GSDME (Rb, 1:1000; Abcam, ab215191), Pro-Caspase-1 (Rb, 1:1000; Abcam, ab179515), β-actin (Ms, 1:5000; Sigma, A5441). Immunoblots were detected using Amersham Imager 600 system (GE healthcare, Little Chalfont, UK). β-actin was used as an internal standard. At least three independent experiments were performed.

### Mitochondrial membrane potential assay

Depolarization of mitochondrial membrane was detected using tetramethylrhodamine, ethyl ester (TMRE) (Abcam, Cambridge, MA, USA) with the manufacturer’s protocol. Briefly, 3 × 10^3^cells/well were seeded in 96-well plates. After incubation for 24 h at 37 °C, cells were treated with or without tetraarsenic hexoxide for 24 h. Then TMRE (200 nM) was added and incubated at 37 °C for 30 min. The plates were read using a SpectraMAX M2E 384 plate reader (Molecular Devices, San Jose, CA, USA) right after the incubation period. All experiments were repeated three times.

### Immunofluorescence assays

Cells at a density of 2 × 10^4^/well were grown in 6-well glass-bottom plates, and then stained using 20 μM DCFDA (Abcam, Cambridge, MA, USA), 5 μM MitoSox (Invitrogen, Waltham, MS, USA), 2 μM JC-1 dyes following the manufacturer’s protocol. DCFDA fluorescence was detected at 485 nm excitation and 535 nm emission and MitoSox fluorescence were detected at 510 nm excitation and 580 nm emission using Victor3 Multi label counter (PerkinElmer, Shelton, CT, USA). JC-1 fluorescence shifting was detected from red fluorescence to green fluorescence. JC-1 monomers were detected at 529 nm excitation and JC-1 aggregates were detected at 590 nm excitation. JC-1 fluorescence was detected by using LSM800 (Carl Zeiss, Jena, Germany).

### Flow cytometry assay

Live cells were detected by flow cytometry in Hank’s Buffered Salt Solution (HBSS) with 2% bovine serum albumin (BSA) using FACS Aria II (Becton Dickinson, Franklin Lakes, NJ, USA). For evaluation of apoptosis, cells were labeled with the Annexin V-FITC Apoptosis Detection Kit (Komabiotech, Seoul, South Korea) following the manufacturer’s protocol. Briefly, cells were pelleted and resuspended in Annexin V-FITC buffer and incubated for 15 min at room temperature, and then propidium iodide (PI) was added for FACS analysis. Annexin V-FITC fluorescence was detected in FL-1 and PI was detected in FL-2. For DCFDA fluorescence, cells were labeled with the DCFDA Cellular ROS detection assay kit (Abcam, Cambridge, MA, USA) following the manufacturer’s protocol. Briefly, cells were pelleted and stained with 20 μM DCFDA for 30 min at 37 °C in the dark. For MitoSox fluorescence, cells were labeled with MitoSox Red mitochondrial superoxide indicator (Invitrogen, Waltham, MS, USA) in 5 μM concentration for 30 min at 37 °C in the dark. After incubation, cells were trypsinized and washed with HBSS two times and analyzed on FACS. For JC-1 fluorescence, cells were labeled with JC-1 Mitochondrial Potential Sensor (Invitrogen, Waltham, MS, USA) with 2 μM JC-1 for 15 min at 37 °C in the dark. After incubation, cells were trypsinized and washed with cold HBSS and analyzed. JC-1 fluorescence was detected in FL-1 and FL-2, respectively, and the mitochondrial depolarization was determined by red fluorescence/green fluorescence ratio. The experiments were performed in triplicate and analyzed using De Novo FCS Express software (Pasadena, CA, USA).

### Gene silencing using RNAi

The siRNA duplexes were designed and synthesized by Bioneer (Daejeon, South Korea) and used to silence *Stat3* and *Dfna5* siRNA expression. The following mouse-specific siRNAs synthesized were used: mouse *Stat3* siRNA #1 (5′-CACAGUUCCUGCACCUACU-3′), mouse *Stat3* siRNA #2 (5′-GCUCAGGGAGUAUGGUCCU-3′), mouse *Dfna5* siRNA #1 (5′-GAGGAAGAGCUUUGUCAGU-3′), mouse *Dfna5* siRNA #2 (5′-GUCUCACACUUGAACGACU-3′). The following human-specific siRNAs synthesized were used: human *DFNA5* siRNA #1 (5′-GUCUGACCCUUUAAUCCAA-3′), human *DFNA5* siRNA #2 (5′-GAAAUACGAGGGCAAGUUU-3′). For the transfection, the siRNA duplexes were transfected into the cells as a final concentration of 25 μM for each siRNA with Lipofectamine RNAiMAX Reagent (Thermo Fisher, Waltham, MS, USA) for 24 h following the manufacturer’s instructions. After transfection, the transfection medium was replaced with a regular growth medium.

### Immunohistochemistry and TUNEL assay

All tumor and lung tissues were embedded in paraffin for hematoxylin and eosin staining and immunohistochemistry. For immunohistochemistry analysis, Primary tumor sections were fixed and stained using anti-Ki67 (Rb, 1:200; Abcam, #ab16667) and anti-caspase-3 (Rb, 1:200; Cell Signaling, #9662) antibodies and counterstained with hematoxylin. For the visualization of the antibodies, 3,3′-Diaminobenzidine (DAB) (Vector Lab, Burlingame, CA, USA) was used. For TUNEL assay, tumor tissues were stained using DeadEnd Fluorometric TUNEL system (Promega, Madison, WI, USA) following the manufacturer’s instructions. Briefly, tumor sections were deparaffinized with xylene and rehydrated gradually using ethanol. Then, tissues were permeabilized with Proteinase K and labeled with Terminal Deoxynucleotidyl Transferase, Recombinant, (rTdT) enzyme. Localized green fluorescent of apoptotic cells were detected using CELENA^®^S Digital Imaging System (Logos Biosystems, Gyeonggi-do, South Korea).

### Reverse transcription and real-time quantitative PCR

Total RNA was isolated from cells using TRIzol reagent (Invitrogen, Waltham, MS, USA) according to the manufacturer’s protocol. Reverse transcription was performed with 1 μg of pure RNA using M-MLV reverse transcriptase (Promega, #M1705, Madison, WI, USA). The synthesized cDNA was amplified by PCR using specific primers. PCR products were visualized by electrophoresis on agarose gels with RedSafe (iNtRON, Gyeonggi-do, South Korea) stain and analyzed using an ImageQuant LAS 4000 image analyzer (GE Healthcare Life Sciences, North Richland Hills, TX, USA). Real-time quantitative PCR using 2× SYBR Green PCR Master Mix (TaKaRa, Kyoto, Japan) was performed by a QuantStudio 5 system (Applied Biosystems, Foster City, CA, USA). All reactions were performed at least three times independently. The following primer sequences were used for RT-PCR: human *APOL6*; Forward: 5′-GCCACCAAAAAGCTACCAAG-3′, Reverse: 5′-GATGCTGTTGACCTGAGCAA-3′, human *18S*; Forward: 5′-AATGCTTCTCTGGCACGTCT-3′, Reverse: 5′-TCTTCCATCTCACGCATCTG-3′, mouse *Apol6*; Forward: 5′-AGGATGACGCTCCTCTGTGT-3′, Reverse: 5′-AGGAGGCTCATCACTCCAGA-3′, mouse *18S*; Forward: 5′-AATGCTTCTCTGGCACGTCT-3′, Reverse: 5′-TCTTCCATCTCACGCATCTG-3′. The following primer sequences were used for real-time quantitative PCR: mouse *Fgfbp1*; Forward: 5′-TCATCCCTCTCCACCCTGTT-3′, Reverse: 5′-GAAGGAGAGCAGGATGAGGC-3′, mouse *lgfbp3*; Forward: 5′-CACTGCCCTCACTCTGCTC-3′, Reverse: 5′-GCGCGCACTGGGACA-3′, mouse *Cxcl1*; Forward: 5′-GGGTGTTGTGCGAAAAGAAGT-3′, Reverse: 5′-CTCCCACACATGTCCTCACC-3′, mouse *Esm1*; Forward: 5′-CCAGCTGCGAGACATGAAGA-3′, Reverse: 5′- CAATGTTCCGGGCAATCCAC-3′, mouse *Il1α*; Forward: 5′-CGCTTGAGTCGGCAAAGAAA-3′, Reverse: 5′-TGGCAGAACTGTAGTCTTCGT-3′, mouse *Mmp10*; Forward: 5′-ATGGACACTTGCACCCTCAG-3′, Reverse:5′-GGTGGAAGTTAGCTGGGCTT-3′, mouse *Mmp13*; Forward: 5′-TCGCCCTTTTGAGACCACTC-3′, Reverse: 5′-AGCACCAAGTGTTACTCGCT-3′, mouse *Cxcl5*; Forward: 5′-CCCTTCCTCAGTCATAGCCG-3′, Reverse: 5′-CTATGACTTCCACCGTAGGGC-3′, mouse *Apol6*; Forward: 5′-AGCCATCAGACAGAGGAGGA-3′, Reverse: 5′-TCTTCAACATCCAGAGGACTG-3′, mouse *18S*; Forward: 5′-GTAACCCGTTGAACCCCATT-3′, Reverse: 5′-CCATCCAATCGGTAGTAGCG-3′. The mRNA expression levels were calculated using the comparative CT value (2^−ΔΔCT^). All the experiments were repeated three times.

### RNA sequencing

Total RNA from each cell for RNA sequencing was isolated using TRIzol reagent following the manufacturer’s instructions. The total RNA samples were treated with DNase I, purified with miRNeasy Mini Kit (Qiagen, Hilden, Germany) and subsequently examined for quality using an Agilent 2100 bioanalyzer (Agilent, Santa Clara, CA, USA). An Illumina platform (Illumina, San Diego, CA, USA) was used to analyze transcriptomes with a 90 bp paired-end library. Samples were pair-end sequenced with the Illumina HiSeq 2000 platform using HiSeq Sequencing kits. GO and KEGG pathway enrichment analyses were performed using the DAVID tool (http://david.abcc.ncifcrf.gov) and the KEGG orthology-based annotation system (KOBAS) online tool (http://geneontology.org) with cut-off values of *P* < 0.05.

### Quantification and statistical analyses

Statistical significance was calculated using GraphPad Prism 5. For all other comparisons, the two-tailed unpaired Student’s *t*-test was used, and *P* < 0.05 indicated statistical significance. There were no studies in which investigators were blinded, and all experiments were repeated at least three times. No statistical method was used to predetermine sample size. The sample size was chosen on the basis of literature in the field.

## Results

### Tetraarsenic hexoxide induces pyroptotic cell death in TNBC cells

**Fig. 1:**
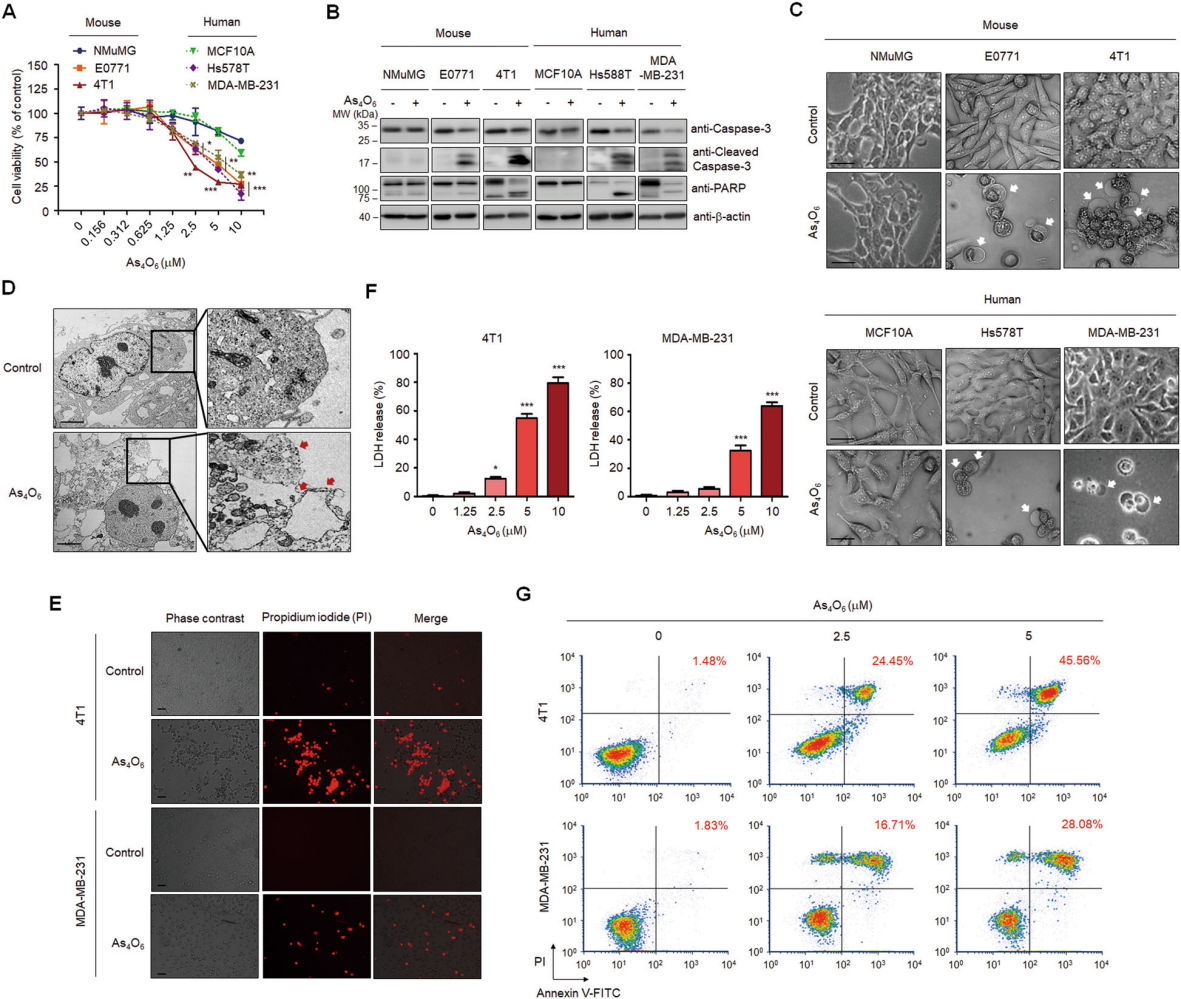
Tetraarsenic hexoxide induces pyroptotic cell death in TNBC cells. **A** Cell doubling times of tetraarsenic hexoxide-treated mouse normal mammary epithelial cells (NMuMG), mouse TNBC cells (EO771, 4T1), human normal-like mammary epithelial cells (MCF10A), and human TNBC cells (Hs578T, MDA-MB-231) cells for 24 h in a dose-dependent manner. *P < 0.05, **P < 0.005, ***P < 0.0005 versus control cells. The data represent the mean ± S.D. of three independent experiments. **B** Representative immunoblot analysis of cleaved caspase-3 and PARP using lysates of tetraarsenic hexoxide-treated cells. β-actin was used as an internal control. Cells were treated with 5 μM tetraarsenic hexoxide for 24 h. **C** Phase-contrast images of tetraarsenic hexoxide-treated cells. Pyroptotic cell morphology was indicated by white arrows. Original magnification, ×200. Scale bar, 50 μm. **D** Representative transmission electron microscopy (TEM) images of 4T1 cells treated with 5 μM tetraarsenic hexoxide for 24 h. Red arrows indicate the large bubbles of the plasma membrane. Scale bar, 2 μm. **E** Fluorescent microscopy images showing propidium iodide (PI) staining in 5 μM tetraarsenic hexoxide-treated TNBC cells for 24 h. Original magnification, ×50. Scale bar, 25 μm. **F** Release of LDH from TNBC cells treated with tetraarsenic hexoxide for 24 h in a dose-dependent manner. *P < 0.05, **P < 0.005, ***P < 0.0005 versus control cells. The data represent the mean ± S.D. of three independent experiments. **G** Flow cytometry analysis of 2.5 and 5 μM tetraarsenic hexoxide-treated TNBC cells for 24 h stained by Annexin V-FITC and PI. The percentage of double-positive cells, which may indicate pyroptotic cells, was labeled in red. All *P* values were calculated by unpaired two-tailed Student’s *t*-tests (**A**, **F**).

Given that patients with TNBC are generally considered incurable with current traditional chemotherapy, highlighting the development of neoadjuvant agents, we first explored the potential anti-tumor effect of tetraarsenic hexoxide in aggressive TNBC cells. To this end, mouse (NMuMG) and human (MCF10A) normal-like mammary epithelial cells, mouse TNBC (EO771, 4T1), and human TNBC (Hs578T, MDA-MB-231) cells were treated with various concentrations of tetraarsenic hexoxide. Interestingly, tetraarsenic hexoxide effectively decreased the cell viability of TNBC cells in a dose-dependent manner, compared to that of normal-like mammary epithelial cells (Fig. 1A). Furthermore, tetraarsenic hexoxide increased expression of cleaved caspase-3 and poly (ADP-ribose) polymerase (PARP), which are pivotal mediators of apoptosis, in TNBC cells without affecting normal-like mammary epithelial cells (Fig. 1B; Supplementary Fig. S1). More strikingly, we found that tetraarsenic hexoxide-treated TNBC cells exhibited microscopic features of cell swelling and balloon-like bubbling, which are morphological features of pyroptotic cells (Fig. 1C). In addition, TEM demonstrated multiple pore formation in the plasma membrane (Fig. 1D). In accordance with this observation, staining with propidium iodide (PI), which is impermeable into cells with the intact plasma membrane, showed that treatment of tetraarsenic hexoxide increased PI fluorescence, compared to control, implying tetraarsenic hexoxide-induced membrane disruption (Fig. 1E). Next, considering that the breakage of plasma membrane integrity induces the release of cytosolic components, we measured the release of lactate dehydrogenase (LDH) as an indication of pyroptotic cell cytotoxicity. Indeed, tetraarsenic hexoxide treatment markedly increased the release of LDH into the cell supernatant in a dose-dependent manner (Fig. 1F). Furthermore, secondary necrosis was observed in the cells undergoing tetraarsenic hexoxide-induced cell death by analyses of flow cytometry of double positive for Annexin V and PI (Fig. 1G). These data suggest that tetraarsenic hexoxide may specifically induce pyroptotic cell death by inducing the rupture and leakage of the plasma membrane in TNBC cells.

### Tetraarsenic hexoxide-induced pyroptosis is dependent on caspase-3-mediated cleavage of GSDME

**Fig. 2:**
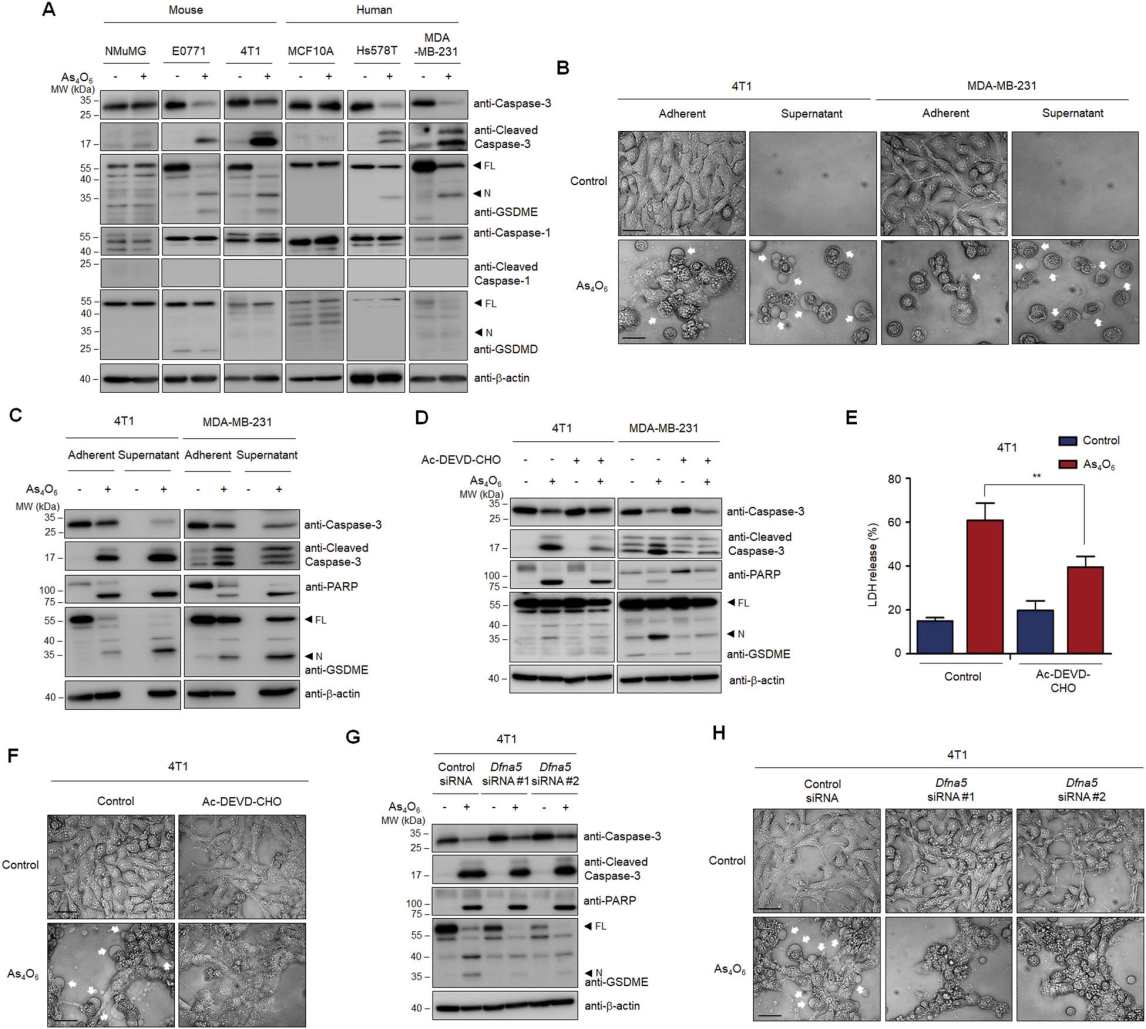
Caspase-3-mediated cleavage of GSDME has involved in tetraarsenic hexoxide-induced pyroptosis in TNBC cells. **A** Representative immunoblot analysis of cleaved caspase-3, PARP, GSDME, caspase-1, and GSDMD in mouse normal mammary epithelial cells (NMuMG), mouse TNBC cells (EO771, 4T1), human normal-like mammary epithelial cells (MCF10A), and human TNBC cells (Hs578T, MDA-MB-231) cells treated with 5 μM tetraarsenic hexoxide for 24 h. β-actin was used as an internal control. **B**, **C** Phase-contrast images (**B**) and representative immunoblot analysis showing cleaved caspase-3, PARP, and GSDME (**C**) from tetraarsenic hexoxide-treated adherent and supernatant TNBC cells. Cells were treated with 5 μM tetraarsenic hexoxide for 24 h, followed by separating adherent and supernatant cells, respectively. **D**–**F** Representative immunoblot analysis of cleaved caspase-3, PARP, and GSDME (**D**) and LDH release (**E**) and phase-contrast images (**F**) from TNBC cells treated with 5 μM tetraarsenic hexoxide for 24 h in the presence or absence of 100 μM Ac-DEVD-CHO. The data represent the mean ± S.D. of three independent experiments. **P < 0.01 using unpaired two-tailed Student’s *t*-tests. **G**, **H** Representative immunoblot analysis (**G**) and phase-contrast images (**H**) of GSDME (encoded by *DFNA5*)-knockdown 4T1 cells upon 5 μM tetraarsenic hexoxide for 24 h. Pyroptotic cell morphology was indicated by white arrows. Original magnification, ×200. Scale bar, 50 μm (**B**, **F** and **H**). FL full length, N N-terminus.

Considering that N-terminal fragments of GSDME cleaved by activation of caspase-3 switch apoptotic cell death to pyroptotic cell death, we initially investigated whether GSDME was involved in tetraarsenic hexoxide-induced pyroptosis. Interestingly, treatment of tetraarsenic hexoxide elevated expression of N-terminal fragments of GSDME with concomitant cleavage of caspase-3 and PARP in TNBC cells without affecting normal-like mammary epithelial cells (Fig. 2A; Supplementary Fig. S2). Notably, N-terminal fragments of GSDMD, which is cleaved by caspase-1, were not observed in TNBC cells. Considering that caspase-3-cleaved GSDME mediates progression to pyroptosis as a secondary necrosis during apoptosis, we speculated that two forms of programmed cell death (PCD) might simultaneously be induced by tetraarsenic hexoxide in TNBC cells. Therefore, we evaluated morphological changes and biochemical markers using adherent cells and the cells from the supernatant exposed to tetraarsenic hexoxide. Upon treatment of tetraarsenic hexoxide, adherent cells underwent morphological changes of apoptosis or pyroptosis as well as cleavage of caspase-3/PARP/GSDME (Fig. 2B, C; Supplementary Fig. S3). However, the cells from the supernatant markedly exhibited increased homogeneous balloon-like bubbling, which represented pyroptotic morphology, and complete fragmentations of caspase-3/PARP/GSDME (Fig. 2B, C; Supplementary Fig. S3). Because both apoptosis and pyroptosis share the same regulatory machinery, such as activation of caspase-3, we examined whether tetraarsenic hexoxide-induced cell death was dependent on the activation of caspase-3. We found that treatment with Ac-DEVD-CHO, a specific inhibitor of caspase-3, decreased the cleavage of caspase-3/PARP/GSDME, the release of LDH, and the apoptotic/pyroptotic phenotypes triggered by tetraarsenic hexoxide (Fig. 2D–F; Supplementary Fig. S4). This result is similar to the previously reported studies showing that chemotherapy drugs-activated caspase-3 cleaves GSDME to induce pyroptosis^20^. We further examined whether GSDME was responsible for tetraarsenic hexoxide-induced pyroptosis in TNBC cells. Interestingly, GSDME knockdown by siRNA did not affect tetraarsenic hexoxide-induced cleavage of caspase-3, compared to that of control siRNA (Fig. 2G; Supplementary Fig. S5). In accordance with this observation, knockdown of GSDME markedly suppressed tetraarsenic hexoxide-induced release of LDH and pyroptotic phenotypes, regardless of the presence of apoptotic phenotypes, implying that caspase-3-cleaved GSDME is required for the induction of pyroptosis by tetraarsenic hexoxide (Fig. 2H; Supplementary Fig. S6). Furthermore, to understand the roles of GSDME in breast cancers, we analyzed the expression of GSDME according to different breast cancer subtypes using public datasets (CCLE dataset; GSE100878; GSE2034). Notably, mRNA expression of GSDME was significantly higher in TNBC cells and the patients with TNBC than in luminal subtypes (Supplementary Fig. S7a–c). Immunoblot analyses supported the public dataset (Supplementary Fig. S7d, e). However, expression of GSDMD was not correlated with breast cancer subtypes (Supplementary Fig. S7d, e). Taken together, these results suggest that tetraarsenic hexoxide induces caspase-3/GSDME-dependent pyroptosis, in addition to triggering caspase-3/PARP-mediated apoptosis, in TNBC cells.

### Tetraarsenic hexoxide promotes ROS-mediated pyroptosis through reduction of the mitochondrial permeability transition in TNBC cells

**Fig. 3:**
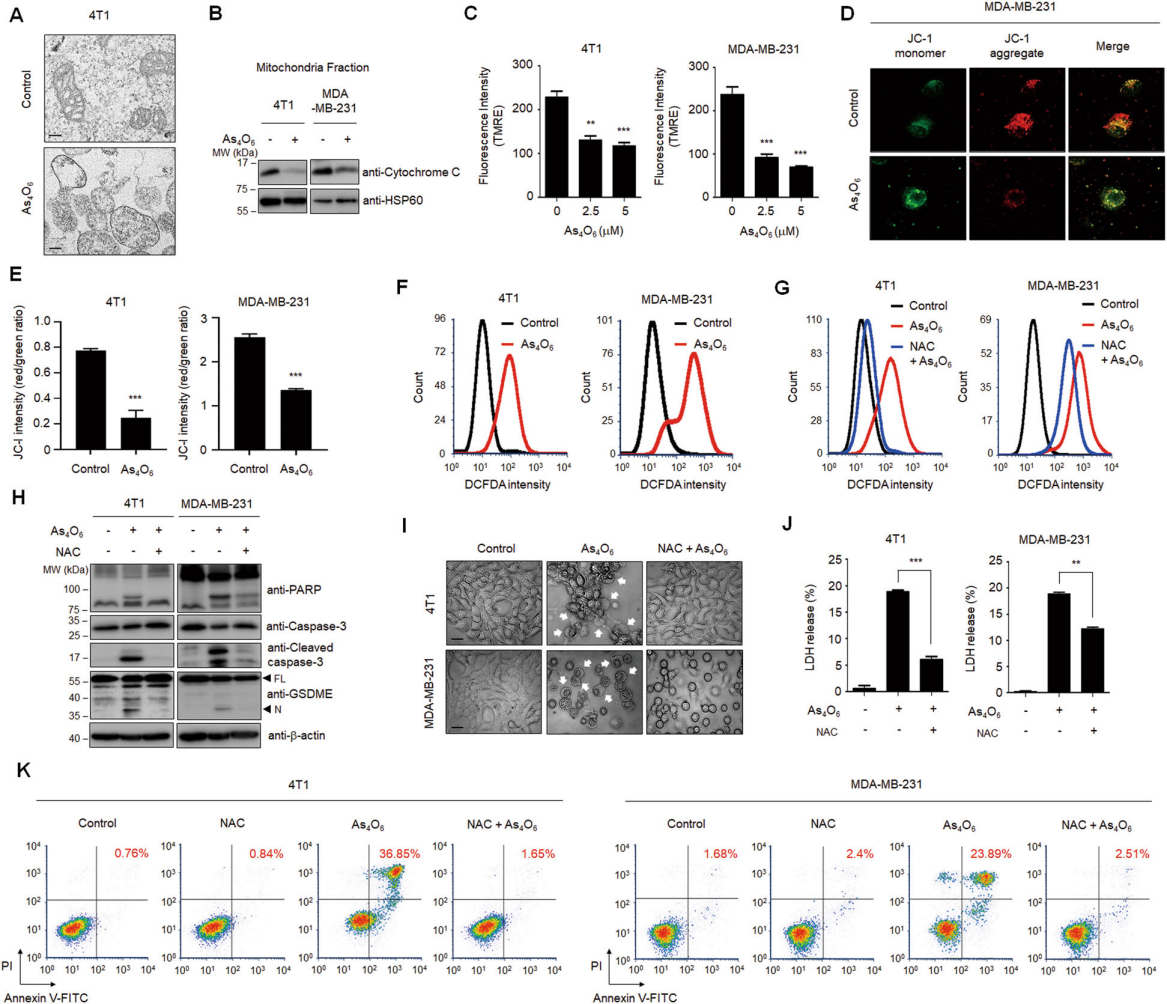
Production of ROS is required for tetraarsenic hexoxide-induced pyroptosis in TNBC cells. **A** Representative transmission electron microscopy images of 4T1 cells treated with 5 μM tetraarsenic hexoxide for 24 h. Scale bar, 200 nm. **B** Representative immunoblot analysis showing cytochrome c expression in mitochondria fractions. Cells were treated with 5 μM tetraarsenic hexoxide for 24 h and then mitochondria were fractionated. HSP60 was used as an internal control of mitochondria fractions. **C**–**E** Quantification of TMRE fluorescence intensity (**C**) and confocal images and quantification of JC-1 dye (**D**, **E**) showing mitochondrial membrane potential in TNBC cells treated with 5 μM tetraarsenic hexoxide for 24 h. **F** Flow cytometry analysis showing cellular ROS levels in TNBC cells. Cells were treated with 5 μM tetraarsenic hexoxide for 24 h and then stained with DCFDA. **G** Cells were pretreated with or without 5 mM NAC for 2 h before treatment of 5 μM tetraarsenic hexoxide for 24 h, and then analyzed using a flow cytometer. **H**–**K** Representative immunoblot analysis (**H**), Phase-contrast images (**I**), LDH release (**J**), and flow cytometry analysis (**K**) showing ROS-mediated pyroptotic characteristics induced by tetraarsenic hexoxide upon pretreatment of NAC in TNBC cells. Cells were pretreated with or without 5 mM NAC for 2 h before treatment of 5 μM tetraarsenic hexoxide for 24 h. Pyroptotic cell morphology was indicated by white arrows. Original magnification, ×200. Scale bar, 50 μm (**I**). The data represent the mean ± S.D. of three independent experiments. ** *P* < 0.01, ***P < 0.001 using unpaired two-tailed Student’s *t*-tests (**C**, **E** and **J**). The data represent the mean ± S.D. of three independent experiments. FL full length, N N-terminus.

We next sought to elucidate the underlying mechanism by which tetraarsenic hexoxide induces GSDME-mediated pyroptosis. Because several studies have shown that GSDME-mediated pyroptosis is closely associated with the mitochondrial pathway, we investigated whether tetraarsenic hexoxide-induced pyroptosis is required for mitochondrial dysfunction. Interestingly, TEM images showed that tetraarsenic hexoxide remarkably increased mitochondrial swelling, compared to the control cells (Fig. 3A). Mitochondrial swelling, which is caused by the disruption of the mitochondrial membrane potential, is often associated with the release of cytochrome c from mitochondria into the cytosol. We found that tetraarsenic hexoxide markedly reduced the expression of cytochrome c in the mitochondrial fraction of TNBC cells (Fig. 3B; Supplementary Fig. S8a). To determine the effect of tetraarsenic hexoxide on the mitochondrial membrane potential, we used TMRE dye, which accumulates in active mitochondria, and JC-1 dye, which shifts from green to red fluorescence in polarized or intact mitochondria. As expected, TMRE intensity was significantly decreased in tetraarsenic hexoxide-treated TNBC cells, compared to the control cells (Fig. 3C). Consistent with this observation, tetraarsenic hexoxide prominently attenuated the ratio of red fluorescent signal as an aggregated dye to green fluorescent signal as a monomeric dye, implying increased mitochondrial depolarization by tetraarsenic hexoxide (Fig. 3D, E). It has been reported that reactive oxygen species (ROS) induces pyroptosis, eventually suppressing cancer progression^24^. Considering that depolarization of mitochondria is closely associated with the generation of ROS, we assumed that tetraarsenic hexoxide might increase levels of cellular ROS. Indeed, flow cytometry analyses by using DCFDA dye, a multiple intracellular ROS indicator, revealed that cellular ROS was significantly increased by treatment of tetraarsenic hexoxide (Fig. 3F; Supplementary Fig. S9a), whereas pretreatment of NAC, a ROS scavenger, decreased cellular ROS levels elevated by tetraarsenic hexoxide (Fig. 3G; Supplementary Fig. S9b). We next examined whether cellular ROS influenced tetraarsenic hexoxide-induced cleavage of caspase-3/PARP/GSDME in TNBC cells, considering that caspase-3-GSDME axis was mainly involved in pyroptosis mediated by cellular ROS. Interestingly, NAC substantially attenuated the cleavage of caspase-3/PARP/GSDME (Fig. 3H; Supplementary Fig. S8b) and pyroptotic characteristics, including the release of LDH, balloon-like bubbling and double positive for Annexin V and PI, upon treatment of tetraarsenic hexoxide, suggesting that cellular ROS mediated tetraarsenic hexoxide-induced pyroptosis by activating caspase-3 and GSDME (Fig. 3I–K). Taken together, these results suggest that tetraarsenic hexoxide increases the generation of cellular ROS through dysfunction of mitochondrial membrane potential, subsequently inducing caspase-3/GSDME-dependent pyroptosis in TNBC cells.

### Tetraarsenic hexoxide induces mitochondrial ROS-mediated pyroptosis by inhibiting phosphorylation of mitochondrial STAT3 in TNBC cells

**Fig. 4:**
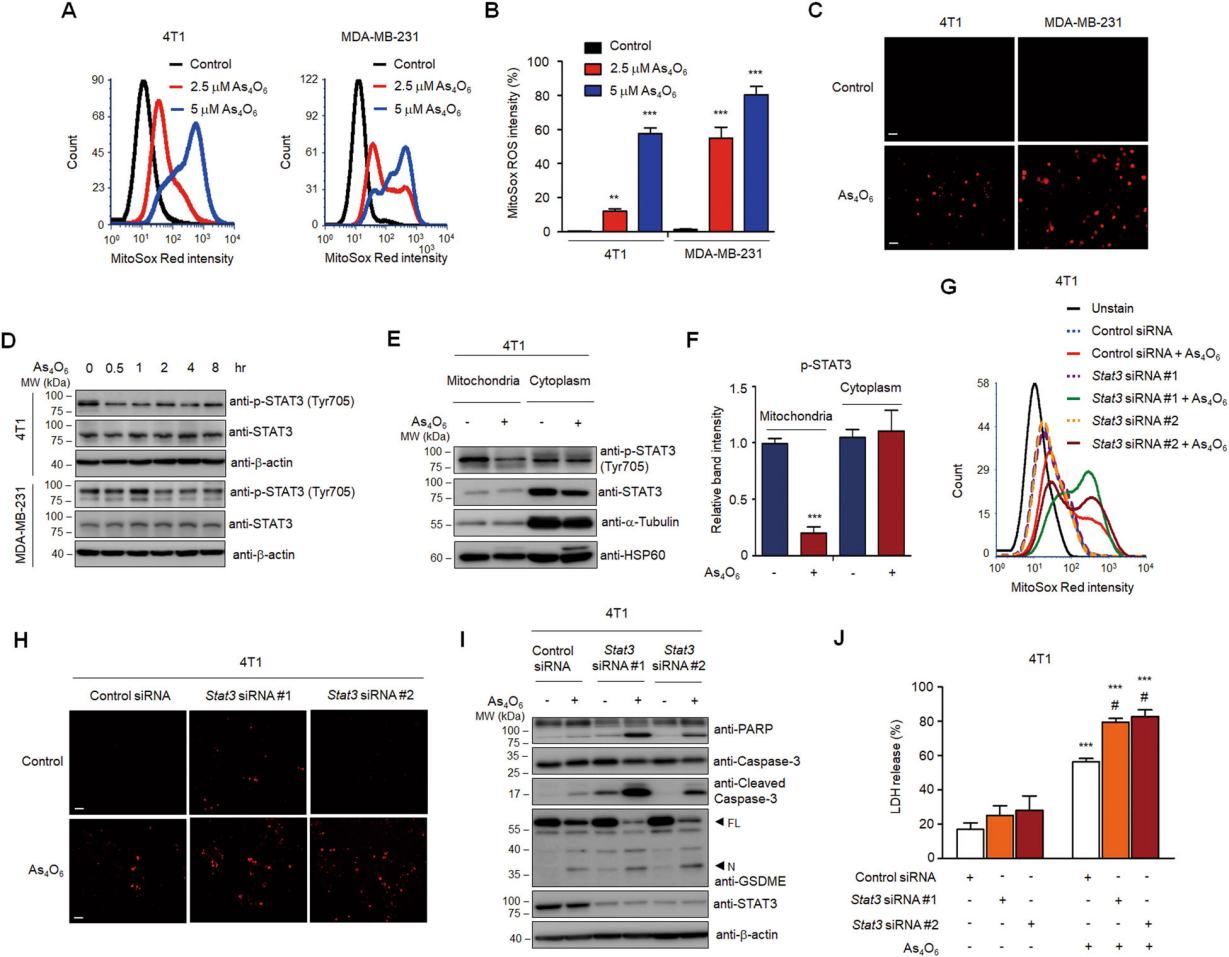
Tetraarsenic hexoxide promotes the production of mitochondrial ROS by inhibiting the phosphorylation of mitochondrial STAT3. **A**, **B** Flow cytometry analysis (**A**) and its quantification (**B**) showing the production of mitochondrial ROS in tetraarsenic hexoxide-treated TNBC cells. Cells were treated with 2.5 and 5 μM tetraarsenic hexoxide for 24 h and then were stained with MitoSox Red. **P < 0.01, ***P < 0.001 versus control cells; **C** Fluorescent microscopy images showing MitoSox Red staining in 5 μM tetraarsenic hexoxide-treated TNBC cells for 24 h. Original magnification, ×50. Scale bar. **D** Representative immunoblot analysis showing the phosphorylation of STAT3 in tetraarsenic hexoxide–treated TNBC cells for the indicated times. **E**, **F** Representative immunoblot analysis (**E**) and densitometric quantitation (**F**) showing the phosphorylation of STAT3 in mitochondria and cytoplasmic fractions. 4T1 cells were treated with 5 μM tetraarsenic hexoxide for 24 h and then mitochondria and cytoplasm were isolated. ***P < 0.0005 versus control cells. **G**, **H** Flow cytometry analysis (**G**) and fluorescent microscopy images (**H**) showing the production of mitochondrial ROS in Stat3-knockdown 4T1 cells. 4T1 cells were transiently transfected with *Stat3* siRNA and then treated with 5 μM tetraarsenic hexoxide for 24 h, followed by staining with MitoSox Red. Original magnification, ×50. Scale bar. i Representative immunoblot analysis showing cleaved caspase-3, PARP, and GSDME from tetraarsenic hexoxide-treated 4T1 cells transiently transfected with *Stat3* siRNA. **J** LDH release by tetraarsenic hexoxide in Stat3-knockdown 4T1 cells. The data represent the mean ± S.D. of three independent experiments. ***P < 0.001 versus tetraarsenic hexoxide-untreated cells; ^#^*P* < 0.01 versus tetraarsenic hexoxide-treated control cells. All *P* values were calculated by unpaired two-tailed Student’s *t*-tests. The data represent the mean ± S.D. of three independent experiments. FL full length, N N-terminus.

Given that mitochondria are a main source of ROS, we further tested whether tetraarsenic hexoxide induces the generation of mitochondrial ROS in TNBC cells. Tetraarsenic hexoxide significantly increased the intensity of MitoSox Red, a selective mitochondrial ROS indicator, in a dose-dependent manner in TNBC cells, indicating that tetraarsenic hexoxide induced the production of mitochondrial ROS (Fig. 4A, B). Consistently, this finding was further supported by an immunofluorescence assay (Fig. 4C). In particular, several studies have reported that signal transducer and activator of transcription 3 (STAT3) is closely linked to the control of the electron transport chain as well as the modulation of mitochondrial ROS in mitochondria^25^. Also, STAT3 is frequently hyper-phosphorylated in TNBC cells than in luminal subtypes^26^. Based on these facts, we assumed that tetraarsenic hexoxide might inhibit phosphorylation of STAT3 to induce the generation of mitochondrial ROS. To this end, we initially confirmed the phosphorylation level of STAT3 upon treatment of tetraarsenic hexoxide in TNBC cells. Interestingly, tetraarsenic hexoxide markedly inhibited constitutively activated phosphorylation of STAT3 in a time-dependent manner (Fig. 4D; Supplementary Fig. S10a). To examine this observation more precisely, we investigated the phosphorylation of STAT3 in the mitochondria fractions and the cytosol fractions of tetraarsenic hexoxide-treated TNBC cells. Strikingly, tetraarsenic hexoxide significantly inhibited STAT3 phosphorylation in the mitochondria fractions, compared to the cytosol fractions, suggesting that tetraarsenic hexoxide specifically suppressed phosphorylation of mitochondrial STAT3 in TNBC cells (Fig. 4E, F). Considering that the generation of mitochondrial ROS is suppressed by mitochondrial STAT3 in a context-dependent manner, we next determined whether the expression of STAT3 regulates tetraarsenic hexoxide-induced production of mitochondrial ROS in TNBC cells. Notably, siRNA-induced STAT3 knockdown markedly enhanced the further intensity of MitoSox Red upon treatment of tetraarsenic hexoxide, compared to those of tetraarsenic hexoxide-treated control cells (Fig. 4G, H). In addition, because it is also reported that increased production of ROS inhibits the phosphorylation of STAT3^27^, we tested whether the ROS generated by tetraarsenic hexoxide suppresses phosphorylation of STAT3. Tetraarsenic hexoxide consistently inhibited phosphorylation of STAT3 in TNBC cells, whereas the decreased phosphorylation was not affected by pretreatment of NAC (Supplementary Fig. S11). Thus, tetraarsenic hexoxide may induce the production of mitochondrial ROS by inhibiting phosphorylation of mitochondrial STAT3. We further examined whether the expression of STAT3 influenced tetraarsenic hexoxide-induced pyroptosis in TNBC cells. Knockdown of STAT3 significantly enhanced tetraarsenic hexoxide-induced cleavage of caspase-3/PARP/GSDME as well as the releases of LDH, compared to those of tetraarsenic hexoxide-treated control cells (Fig. 4I, J; Supplementary Fig. S10b). Collectively, these results suggest that tetraarsenic hexoxide promotes pyroptosis via the generation of mitochondrial ROS by inhibiting phosphorylation of mitochondrial STAT3 in TNBC cells.

### Tetraarsenic hexoxide suppresses tumor growth and lung metastasis in TNBC cells

**Fig. 5:**
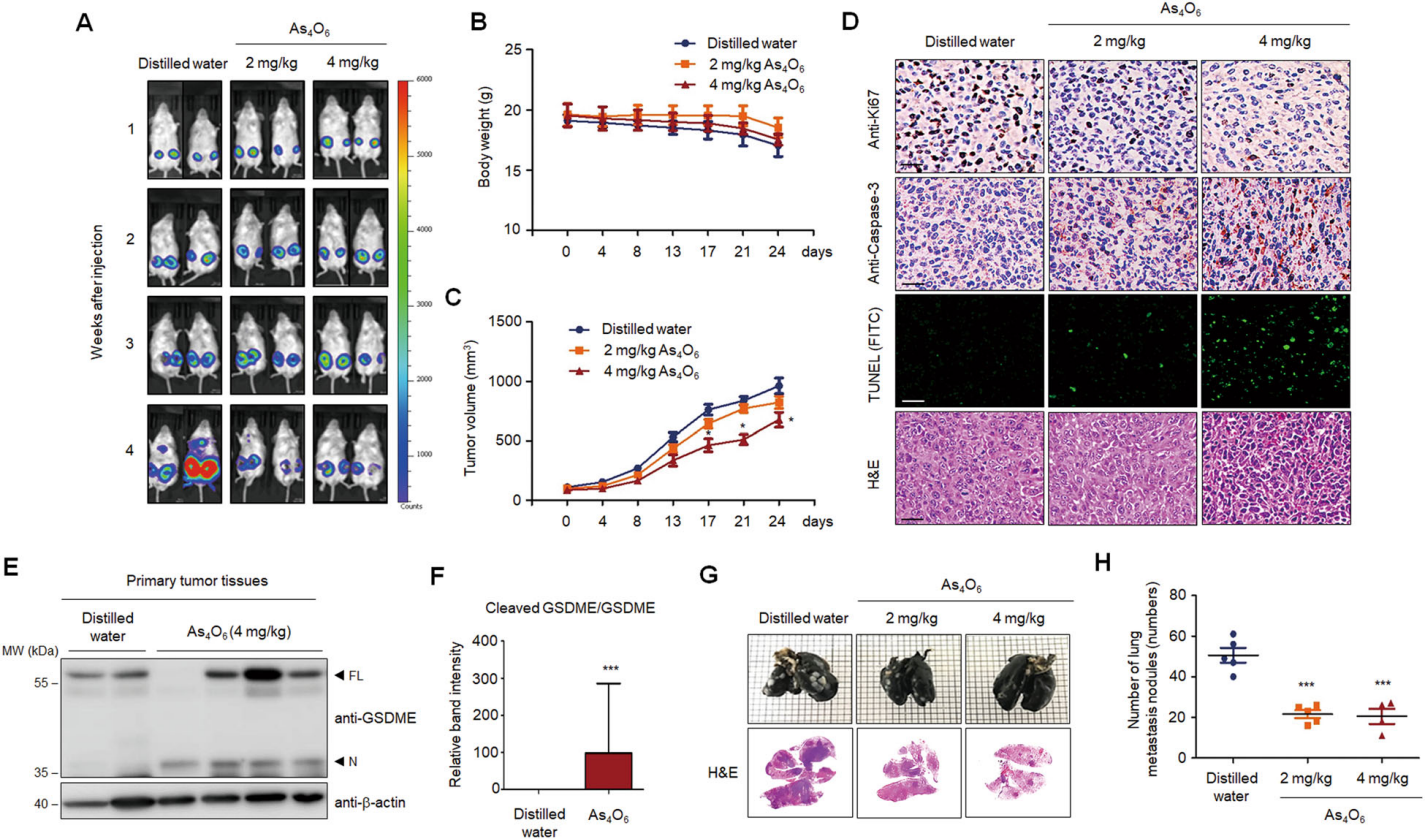
Tetraarsenic hexoxide significantly decreases the primary tumor and spontaneous lung metastasis in aggressive breast cancer cells. **A** Representative Bio Layer Interferometry (BLI) imaging of Balb/c mice showing primary tumors and spontaneous lung metastasis generated by 4T1-Luc cells upon administration of tetraarsenic hexoxide. **B**, **C** Body weight (**B**) and tumor volume (**C**) curves in tetraarsenic hexoxide-administrated 4T1-injected mice. **D** Representative IHC images showing Ki67, caspase-3, and TUNEL in primary tumor tissues from (**A**). Original magnification, ×100. Scale bar, 50 μm. **E**, **F** Representative immunoblot analysis (**E**) and densitometric quantitation (**F**) showing the cleavage of GSDME in primary tumor tissues from (**A**). **G** Representative whole-lung images stained with India ink (upper) and H&E (lower) from (**A**). **H** Scatter plot showing number of lung metastatic nodules from (**A**). *P < 0.05, ***P < 0.0005 versus tetraarsenic hexoxide-treated mice. All *P* values were calculated by unpaired two-tailed Student’s *t* tests (**C**, **F**, **H**). The data represent the mean ± S.D. FL full length, N N-terminus.

Our findings led us to verify anti-tumor effects of tetraarsenic hexoxide in cancer progression of TNBC cells. To this end, we first investigated whether tetraarsenic hexoxide suppressed clonogenic potential and cell migration of TNBC cells in vitro. Treatment of tetraarsenic hexoxide significantly reduced foci formation and cell migration of TNBC cells in a dose-dependent manner in vitro (Supplementary Fig. S12a, b). To further examine the anti-tumor effects of tetraarsenic hexoxide on tumorigenesis and spontaneous lung metastasis of TNBC cells in vivo, we orthotopically injected the luciferase-expressing 4T1 cells into the mammary fat pad of Balb/c mice as a syngeneic mouse breast cancer model. After the size of the primary tumor had grown to 1 cm, tetraarsenic hexoxide was administered daily via intraperitoneal injection in a dose-dependent manner, followed by detection of bioluminescent signal every week. As shown in Fig. 5A, administration of tetraarsenic hexoxide markedly attenuated bioluminescent signal of primary tumor sites as well as metastatic sites in the lung, compared to those in the control group. In accordance with this observation, tetraarsenic hexoxide at 4 mg/kg dose significantly decreased the ability of 4T1 cells to form primary tumor without affecting body weight, compared to the control group (Fig. 5B, C). Furthermore, expression of Ki-67, a marker of cell proliferation, was decreased, whereas expression of active caspase-3 and signal of TUNEL staining was significantly increased in the primary tumor tissues from tetraarsenic hexoxide-administered mice, compared with the control tissues (Fig. 5D). We then tested whether anti-tumor effects of tetraarsenic hexoxide are associated with pyroptosis. Increased cleavage of N-terminal fragment of GSDME was markedly observed in the primary tumor tissues obtained from tetraarsenic hexoxide-administered mice, compared with those from the control mice (Fig. 5E, F). In addition, we further observed that tetraarsenic hexoxide administration resulted in a significant reduction in spontaneous lung metastasis (Fig. 5G, H). Taken together, these results indicate that tetraarsenic hexoxide exerts the anti-tumor effects via GSDME-mediated pyroptosis in aggressive TNBC cells.

### Tetraarsenic hexoxide significantly decreases the expression of cancer progression-related genes in TNBC cells

**Fig. 6:**
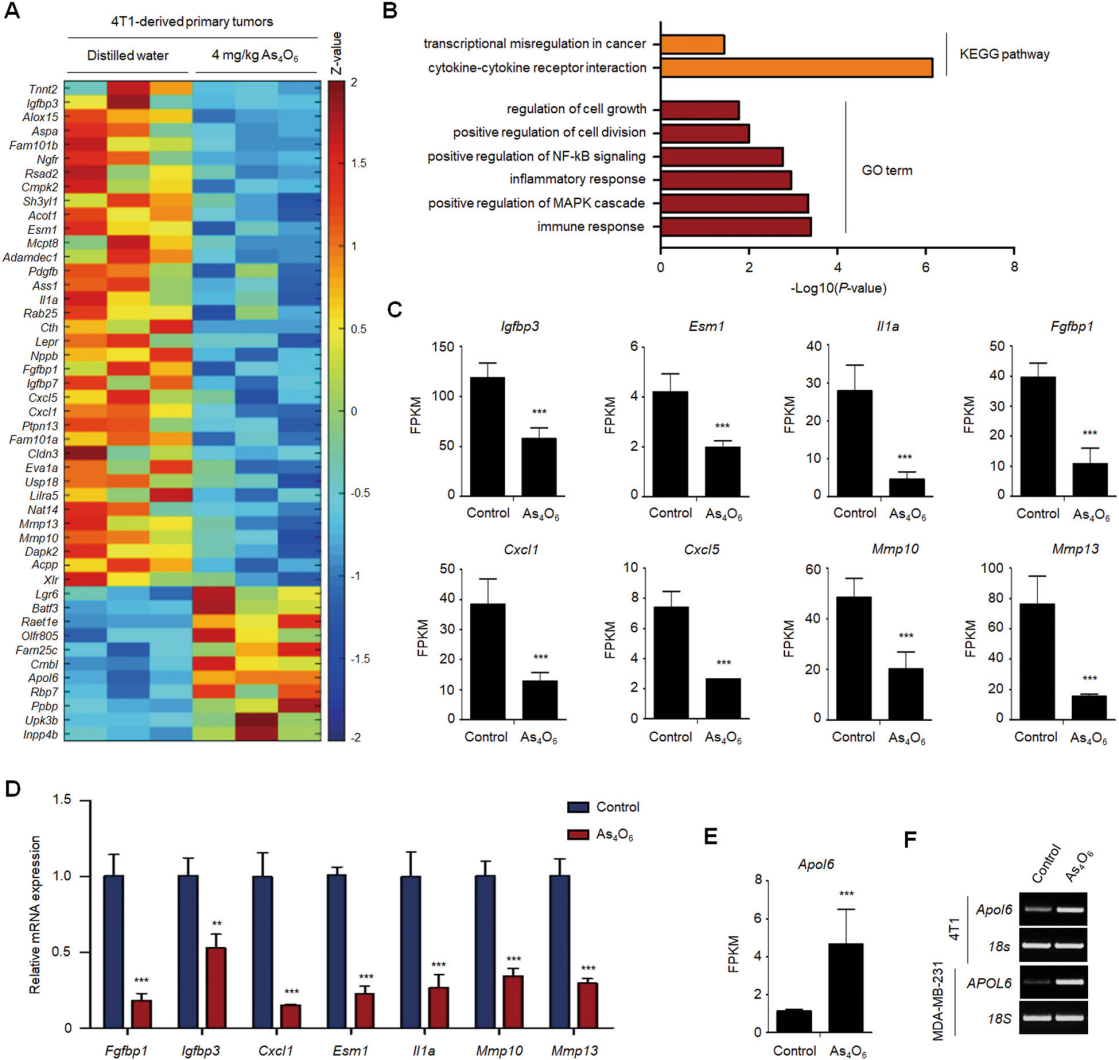
Tetraarsenic hexoxide regulates the expression of cancer progression-associated genes in TNBC cells. **A** Heatmap showing the upregulated and downregulated genes in primary tumor tissue. Threshold values are as follows: corrected value *P* < 0.05 and absolute log2 fold-change (log2FC) > 1.0. **B** KEGG pathways and GO terms enriched in differentially expressed genes (DEGs) from (**A**). **C**, **D** FPKM values and real-time qRT-PCR showing tumor growth-related downregulated genes from (**A**). **E**, **F** FPKM value and RT-PCR showing expression of *APOL6*, a tumor suppressor gene in TNBC cells. **P < 0.01, ***P < 0.001 using unpaired two-tailed Student’s *t*-tests (**C**–**E**). The data represent the mean ± S.D. of three independent experiments.

Based on these in vivo results, to further verify whether tetraarsenic hexoxide regulates the expression of cancer progression-associated genes in TNBC cells, we performed transcriptome analysis using 4T1-derived primary tumor tissues acquired from Fig. 5. A heatmap revealed that tetraarsenic hexoxide administration resulted in a significant decrease of the genes enriched in cancer progression, compared to those of control tumor tissues (with a twofold cutoff, *P* < 0.05) (Fig. 6A). To gain further insights into the genes downregulated in the primary tumor tissues administered with tetraarsenic hexoxide, we analyzed Gene Ontology (GO) terms and Kyoto Encyclopedia of Genes and Genomes (KEGG) pathways utilizing the Database for Annotation, Visualization and Integrated Discovery (DAVID) functional annotation tool. The genes that were downregulated by tetraarsenic hexoxide, including *Igfbp3*, *Esm1*, *Il1a*, *Fgfbp1*, *Cxcl1*, *Cxcl5*, *Mmp10*, and *Mmp13*, which have been implicated in cancer progression, were highly involved in cell growth and transcriptional misregulation in cancer, compared to those in the control tissues (Fig. 6B, C). These findings were further supported by quantitative RT-PCR (Fig. 6D). Interestingly, we also identified that *Apol6*, which induces mitochondria-mediated cell death, was up-regulated in the primary tumor tissues administered with tetraarsenic hexoxide (Fig. 6E), and its expression was also increased by treatment of tetraarsenic hexoxide in TNBC cells (Fig. 6F). Taken together, these results indicate that, besides the induction of GSDME-mediated pyroptosis, alteration of cancer progression-related genes by tetraarsenic hexoxide may be associated with the anti-tumor effects in TNBC cells.

## Discussion

**Fig. 7:**
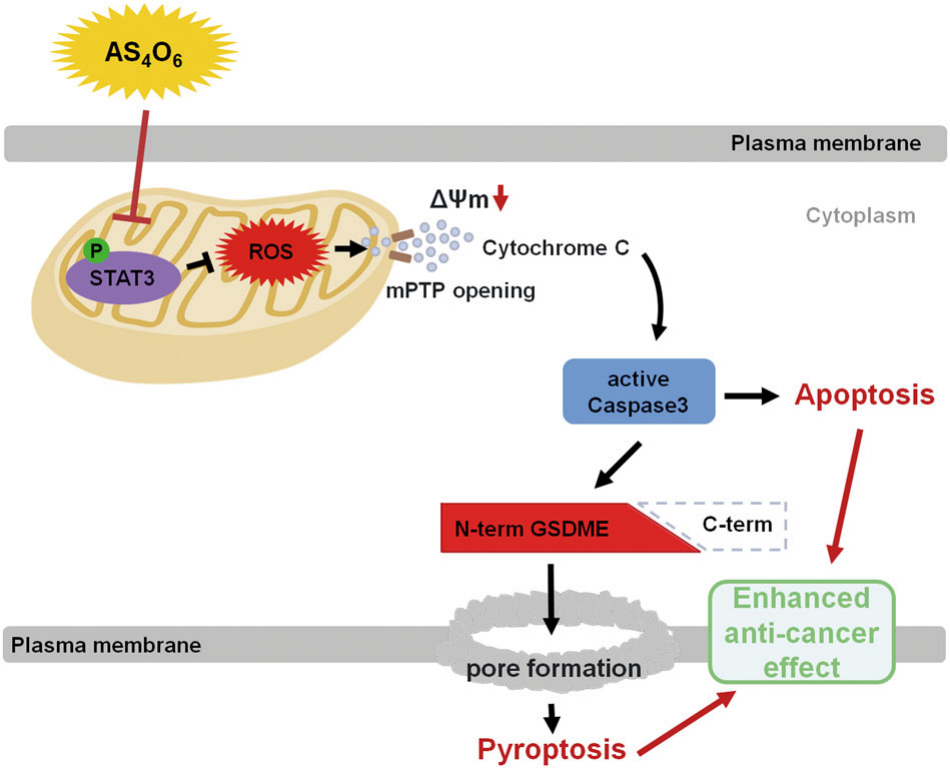
Schematic models demonstrating the anti-tumor effect of tetraarsenic hexoxide in TNBC cells. Tetraarsenic hexoxide promotes pyroptosis through increased production of mitochondrial ROS by inhibiting the phosphorylation of mitochondrial STAT3, subsequently triggering the cleavage of caspase-3/GSDME, eventually suppressing cancer progression of TNBC cells. mPTP mitochondrial permeability transition pore.

In this study, our findings have demonstrated the anti-tumor effect of tetraarsenic hexoxide, suggesting it as a potential new chemotherapeutic agent for TNBC therapy. Notably, we found that tetraarsenic hexoxide induces typical pyroptotic characteristics, including balloon-like bubbling and release of LDH through pore formation in the plasma membrane in TNBC cells. Furthermore, we proposed a mechanism by which tetraarsenic hexoxide induced pyroptotic cell death via mitochondrial ROS-mediated caspase-3/GSDME pathway by inhibiting phosphorylation of mitochondrial STAT3, thereby suppressing tumor growth and metastatic potential of aggressive TNBC cells (Fig. 7).

Although it has long been known that apoptosis is a potent mechanism for tumor suppression following treatment with various anti-tumor agents, there is an increasing number of studies related to other forms of PCD, especially pyroptosis, through activation of specific signaling pathways in various tumor cells. Pyroptosis, an inflammatory form of the final phase of PCD, has been considered as GSDMD-mediated cell death through activation of inflammatory caspases (caspase-1/4/5/11) in immune cells^28,29^. In addition, recent studies showed that chemotherapeutic or molecular targeted agents-induced activation of caspase-3 triggers pyroptosis by cleaving GSDME specifically, resulting in a decrease of cell growth in various cancer cells^20,30^. However, given that various chemotherapeutic agents currently used in clinical cancer therapy are often associated with chemoresistance or side effects, there is a need to develop effective neoadjuvant chemotherapy that can improve the poor prognosis of patients with cancer. Although previous studies have mainly focused on the anti-tumor effect of tetraarsenic hexoxide on apoptosis, we first found that tetraarsenic hexoxide markedly induced pyroptotic cell death through caspase-3-mediated cleavage of GSDME in TNBC cells. Indeed, tetraarsenic hexoxide-treated TNBC cells exhibited the pyroptotic cell death characterized by cell swelling, balloon-like bubbling, the rupture of the plasma membrane, and the release of LDH. Moreover, consistent with the important role of caspase-3 in releasing N-terminal fragments of GSDME, which consequently leads to pore formation of the plasma membrane during pyroptotic cell death, tetraarsenic hexoxide significantly induced the cleavage of caspase-3 as well as GSDME, increasing the release of LDH. Supporting this observation, inhibition of active caspase-3 attenuated tetraarsenic hexoxide-induced pyroptotic features by blocking the cleavage of GSDME. Furthermore, tetraarsenic hexoxide markedly attenuated the tumorigenic and metastatic capacity of TNBC cells. Considering that GSDMD also induces pyroptosis, it is feasible that the cleavage of GSDMD is required to induce pyroptosis by tetraarsenic hexoxide in TNBC cells. However, interestingly, tetraarsenic hexoxide did not affect the cleavage of GSDMD. In addition, considering that GSDME was overexpressed in TNBC cells compared with luminal subtype cells, which are non-aggressive breast cancer cells, tetraarsenic hexoxide-induced pyroptosis may be dependent on the GSDME expression level in breast cancer cells. Collectively, tetraarsenic hexoxide-triggered pyroptosis may specifically be dependent on the caspase-3-mediated cleavage of GSDME in TNBC cells.

Our findings of the current study raise questions regarding how tetraarsenic hexoxide induces pyroptosis through the cleavage of caspase-3/GSDME in TNBC cells. Previous studies have been reported that ROS plays an important role in pyroptosis. For example, lobaplatin induced caspase-3/GSDME-mediated pyroptosis by increasing cellular ROS levels in colon cancer cells^24^. Moreover, iron-elevated ROS induced pyroptosis through activation of Bax/caspase-3/GSDME pathway by facilitating the oxidation of mitochondrial outer membrane protein Tom20 in melanoma cells^31^. Based on these reports, we speculated that tetraarsenic hexoxide might induce pyroptosis by increasing ROS levels in TNBC cells. Our results suggested that tetraarsenic hexoxide markedly elevates the generation of cellular ROS by increasing mitochondrial depolarization to induce caspase-3/GSDME-mediated pyroptosis in TNBC cells. Indeed, tetraarsenic hexoxide reduced the mitochondrial membrane potential and increased the cellular ROS levels, whereas the addition of antioxidant N-acetyl Cysteine (NAC) markedly decreased cellular ROS levels elevated by tetraarsenic hexoxide, consequently blocking the cleavage of caspase-3/GSDME and pyroptotic characteristics. Furthermore, although the correlation between STAT3 and ROS is still controversial, it is reported that mitochondrial STAT3 is associated with the generation of mitochondrial ROS by regulating the mitochondrial permeability transition pore^32^. Mitochondrial STAT3 limited the production of mitochondrial ROS in response to stress insult^33^, and knockdown of STAT3 increased the generation of cellular ROS in TNBC and pancreatic cancer cells^27,34^. In this regard, it is possible that tetraarsenic hexoxide might induce the production of mitochondrial ROS through inhibition of mitochondrial STAT3 activation. Indeed, tetraarsenic hexoxide markedly decreased phosphorylation of STAT3 and increased the production of mitochondrial ROS in TNBC cells. In addition, STAT3 knockdown enhanced tetraarsenic hexoxide-induced production of mitochondrial ROS, cleavage of caspase-3/GSDME, and the releases of LDH. Therefore, we reasoned that tetraarsenic hexoxide induces mitochondrial ROS-mediated pyroptosis by targeting mitochondrial STAT3 in TNBC cells. Further comprehensive work is necessary to understand how tetraarsenic hexoxide regulates the activation of mitochondrial STAT3 in mitochondria.

Although GSDME is known as a tumor suppressor, it is reported that GSDME is highly expressed in several normal tissues and chemotherapy toxicity influences GSDME-mediated pyroptosis, eventually leading to the damage of normal cells^20^. In the case of our study, no side effects were observed in the mice administered with tetraarsenic hexoxide as well as in NMuMG normal mouse mammary epithelial cells upon tetraarsenic hexoxide treatment, indicating that tetraarsenic hexoxide exerts specific effects in TNBC cells, but not in normal cells. It could be explained that the level of cellular ROS is commonly increased in cancer cells because of their fundamental defects in the oxidative metabolism system, while oxidative stress is rapidly neutralized by the antioxidant defense system in normal cells. Thus, TNBC cells may be more sensitive to tetraarsenic hexoxide-induced accumulation of ROS compared with normal cells. Further comprehensive work is needed to gain deeper insight into the anti-tumor effect of tetraarsenic hexoxide on other malignant tumors.

In conclusion, our results suggest the mechanism by which tetraarsenic hexoxide induces pyroptosis through the increase of mitochondrial ROS by targeting phosphorylation of mitochondrial STAT3, subsequently activating caspase-3/GSDME that eventually leads to suppression of cancer progression of TNBC cells. In addition, given that aggressive TNBC cells are often resistant to apoptosis triggered by traditional chemotherapeutic agents, tetraarsenic hexoxide may be useful as a new GSDME-targeted therapeutic strategy against metastatic breast cancers.

## Supplementary information

Supplementary Figure Legends

Supplementary Figure S1

Supplementary Figure S2

Supplementary Figure S3

Supplementary Figure S4

Supplementary Figure S5

Supplementary Figure S6

Supplementary Figure S7

Supplementary Figure S8

Supplementary Figure S9

Supplementary Figure S10

Supplementary Figure S11

Supplementary Figure S12
