# Supplementary material for: Tetraarsenic hexoxide enhances generation of mitochondrial ROS to promote pyroptosis by inducing the activation of caspase-3/GSDME in triple-negative breast cancer cells
Source: Cell Death Dis. 2021 Feb 8;12(2):159. doi: 10.1038/s41419-021-03454-9 (PMC7870965; doi:10.1038/s41419-021-03454-9)
Supplement: Supplementary file 1 — Supplementary Figure Legends [file 41419_2021_3454_MOESM1_ESM.docx]

**Supplementary Figure legends**

**Tetraarsenic hexoxide enhances generation of mitochondrial ROS to promote pyroptosis by inducing the activation of caspase-3/GSDME in triple-negative breast cancer cells**

Haein An, Jin Sun Heo, Pyunggang Kim, Zenglin Lian, Siyoung Lee, Jinah Park, Eunji Hong, Kyoungwha Pang, Yuna Park, Akira Ooshima, Jihee Lee, Minjung Son, Hyeyeon Park, Zhaoyan Wu, Kyung-Soon Park, Seong-Jin Kim, Illju Bae, and Kyung-Min Yang

**Supplementary Figure S1** Densitometric quantitation of immunoblot bands for **Figure 1B**. **P*<0.05, ***P*<0.005, ****P*<0.0005 versus control cells. All *P* values were calculated by unpaired two-tailed Student’s *t*-tests. The data represent the mean ± S.D. of three independent experiments.

**Supplementary Figure S2** Densitometric quantitation of immunoblot bands for **Figure 2A**. ***P*<0.005, ****P*<0.0005 versus control cells. All *P* values were calculated by unpaired two-tailed Student’s *t*-tests. The data represent the mean ± S.D. of three independent experiments.

**Supplementary Figure S3** Densitometric quantitation of immunoblot bands for **Figure 2C**. ***P*<0.005, ****P*<0.0005 versus control cells. All *P* values were calculated by unpaired two-tailed Student’s *t*-tests. The data represent the mean ± S.D. of three independent experiments.

**Supplementary Figure S4** Densitometric quantitation of immunoblot bands for **Figure 2D**. ***P*<0.005, ****P*<0.0005 versus control cells. All *P* values were calculated by unpaired two-tailed Student’s *t*-tests. The data represent the mean ± S.D. of three independent experiments.

**Supplementary Figure S5** Densitometric quantitation of immunoblot bands for **Figure 2G**. ****P*<0.0001 versus control cells. All *P* values were calculated by unpaired two-tailed Student’s *t*-tests. The data represent the mean ± S.D. of three independent experiments.

**Supplementary Figure S6** Release of LDH from GSDME knockdown 4T1 cells treated with 5 μM tetraarsenic hexoxide for 24 h. The data represent the mean ± S.D. of three independent experiments. **P*<0.05, ***P*<0.005 using unpaired two-tailed Student’s *t*-tests.

**Supplementary Figure S7** GSDME is highly expressed in aggressive breast cancer cells. (**a-c**) Expression of *GSDME* (encoded by *DFNA5*) in different subtypes of breast cancer cells and patient tissues obtained from CCLE database, GSE100878, and GSE2034. (**d, e**) Representative immunoblot analysis (**d**) and densitometric quantitation (**e**) showing expression of GSDME in breast cancer cells.

**Supplementary Figure S8** Densitometric quantitation of immunoblot bands for **Figure 3B** (**a**) **and 3H** (**b**). ****P*<0.0001 using unpaired two-tailed Student’s *t*-tests. The data represent the mean ± S.D. of three independent experiments.

**Supplementary Figure S9** Tetraarsenic hexoxide markedly increases the generation of cellular ROS levels in TNBC cells. (**a**) Fluorescent microscopy images showing cellular ROS levels in 4T1 cells. Cells were treated with 2.5 and 5 μM tetraarsenic hexoxide for 24 h and then stained with DCFDA. (**b**) 4T1 cells were pretreated with or without 5 mM NAC for 2 h before treatment of 5 μM tetraarsenic hexoxide for 24 h and then stained with DCFDA. Original magnification, 50×. Scale bar.

**Supplementary Figure S10** Densitometric quantitation of immunoblot bands for **Figure 4D** (**a**) **and 4I** (**b**). **P*<0.05, ***P*<0.005, ****P*<0.0005; #*P* < 0.05, #*P* < 0.005 versus tetraarsenic hexoxide-treated control cells using unpaired two-tailed Student’s *t*-tests. The data represent the mean ± S.D. of three independent experiments.

**Supplementary Figure S11** Tetraarsenic hexoxide-induced cellular ROS does not inhibit the phosphorylation of STAT3. Representative immunoblot analysis (**a**) and densitometric quantitation (**b**) showing the phosphorylation of STAT3 in tetraarsenic hexoxide-treated TNBC cells in the presence or absence of NAC. ***P* < 0.005, ****P* < 0.0005 using unpaired two-tailed Student’s *t*-tests. The data represent the mean ± S.D. of three independent experiments.

**Supplementary Figure S12** Tetraarsenic hexoxide significantly decreases the foci formation and cell migration in aggressive breast cancer cells. Focus-forming assay and migration assay of control and tetraarsenic hexoxide treated 4T1 and MDA-MB-231 cells. 1.25 or 2.5 μM of tetraarsenic hexoxide was treated for 5-6 days and foci were stained by methylene blue (**a**). 3 or 6 μM of tetraarsenic hexoxide was treated for 16 h and migrated cells were stained by methylene blue (**b**). **P*<0.05, ****P*<0.005 using unpaired two-tailed Student’s *t*-tests. The data represent the mean ± S.D. of three independent experiments.
