# Supplementary figures and images for: Tetraarsenic hexoxide enhances generation of mitochondrial ROS to promote pyroptosis by inducing the activation of caspase-3/GSDME in triple-negative breast cancer cells
Source: Cell Death Dis. 2021 Feb 8;12(2):159. doi: 10.1038/s41419-021-03454-9 (PMC7870965; doi:10.1038/s41419-021-03454-9)

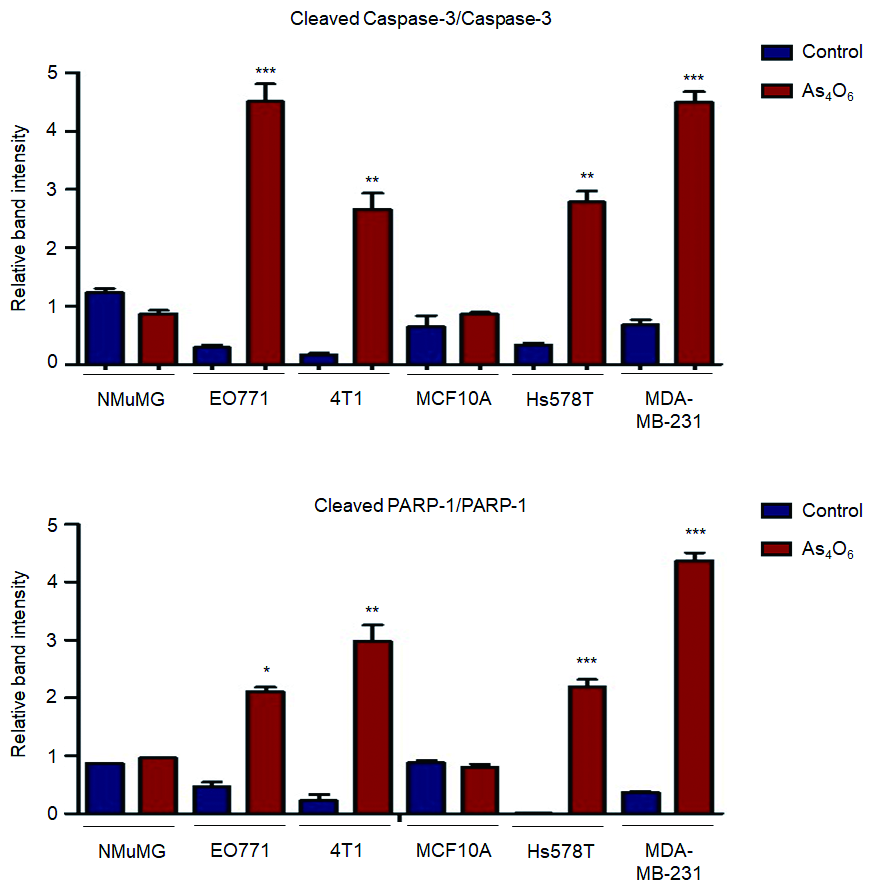

Supplement: Supplementary file 2 — Supplementary Figure S1 [file 41419_2021_3454_MOESM2_ESM.tif]

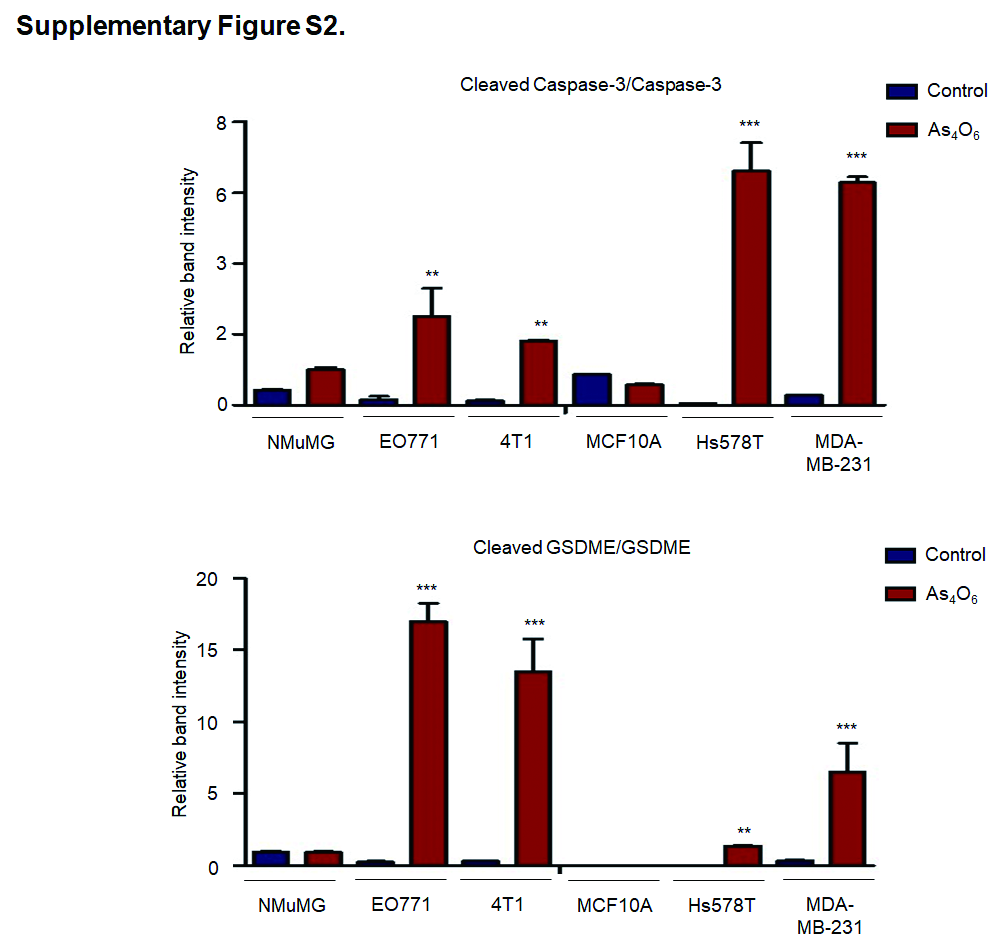

Supplement: Supplementary file 3 — Supplementary Figure S2 [file 41419_2021_3454_MOESM3_ESM.tif]

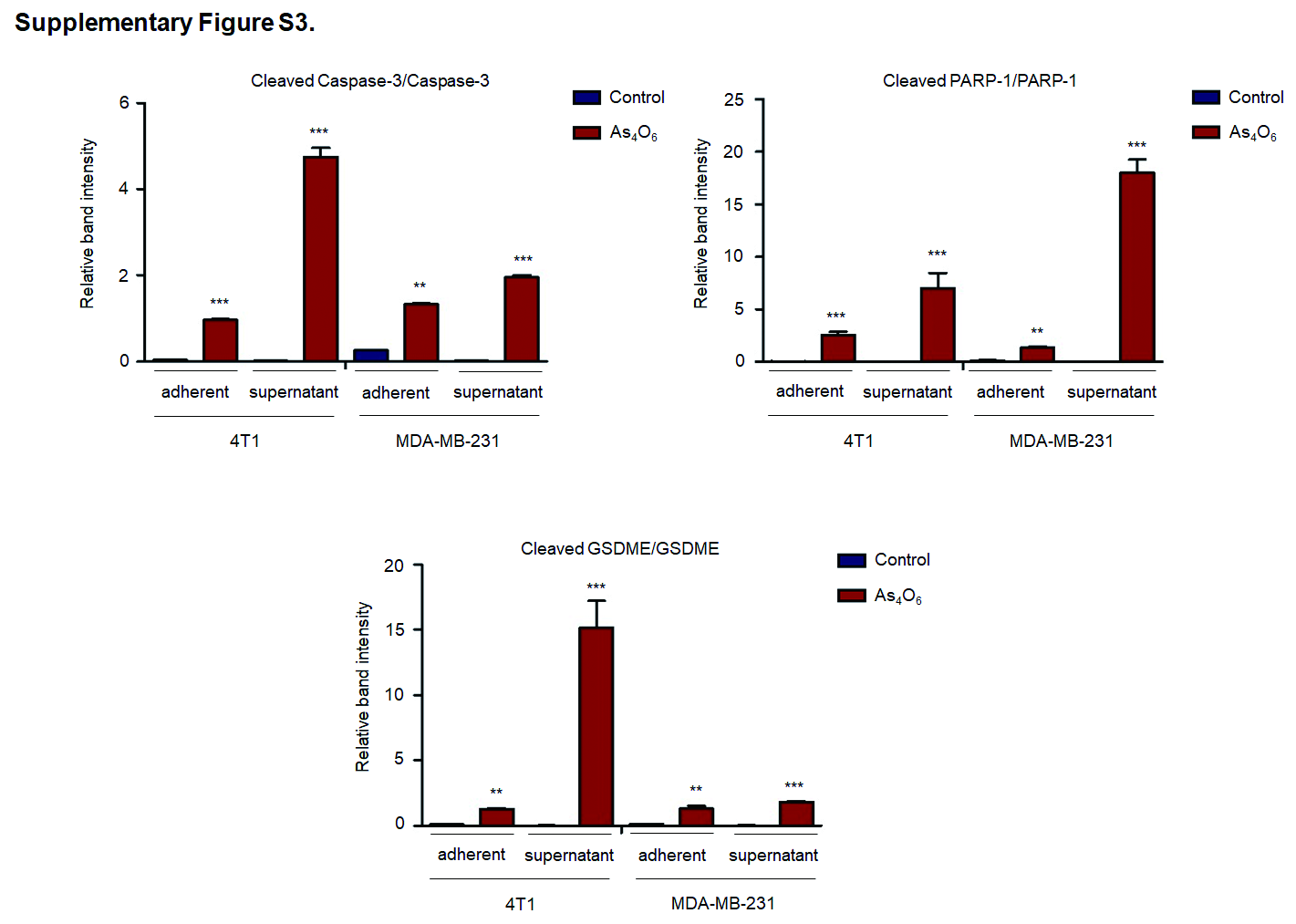

Supplement: Supplementary file 4 — Supplementary Figure S3 [file 41419_2021_3454_MOESM4_ESM.tif]

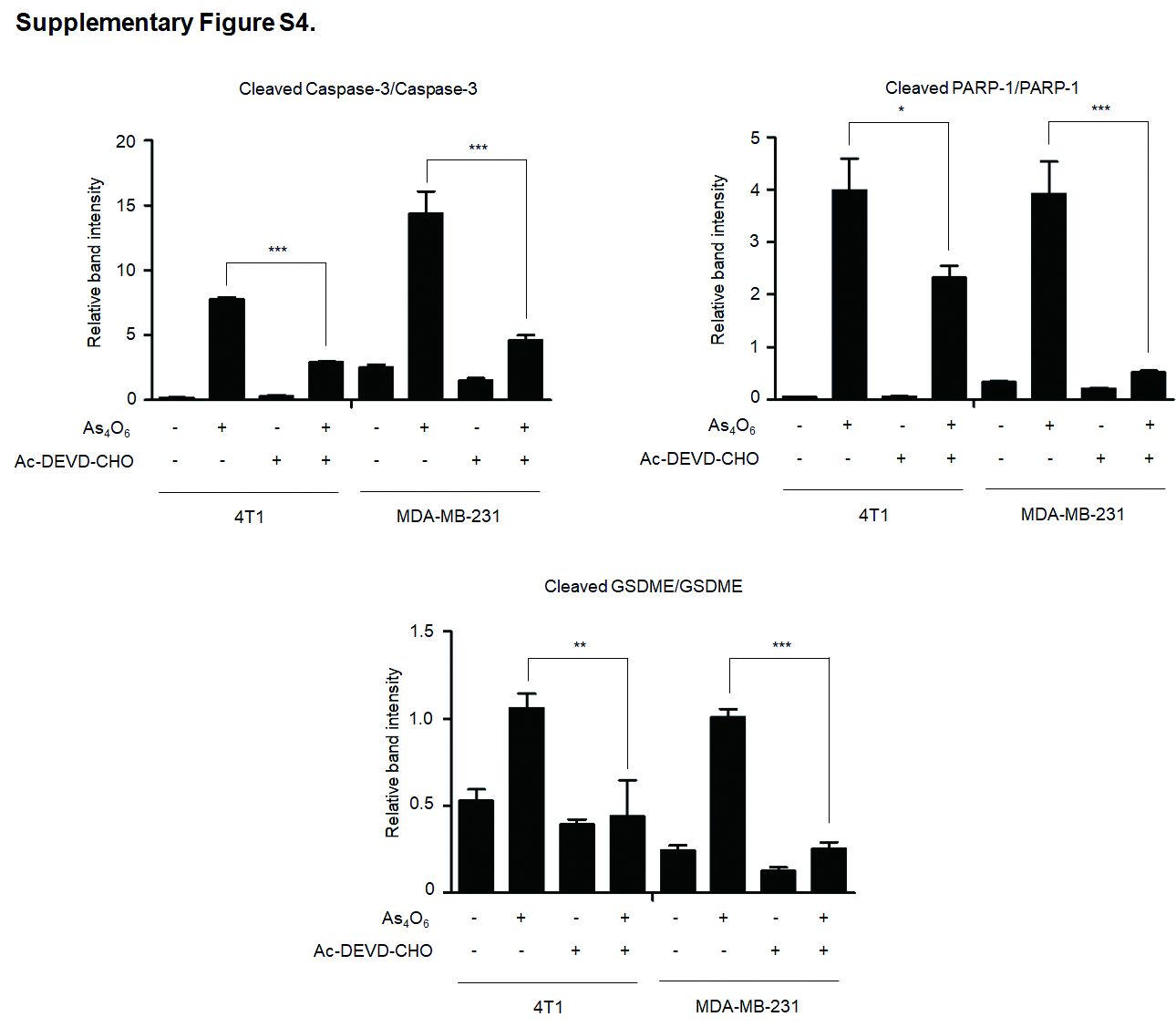

Supplement: Supplementary file 5 — Supplementary Figure S4 [file 41419_2021_3454_MOESM5_ESM.tif]

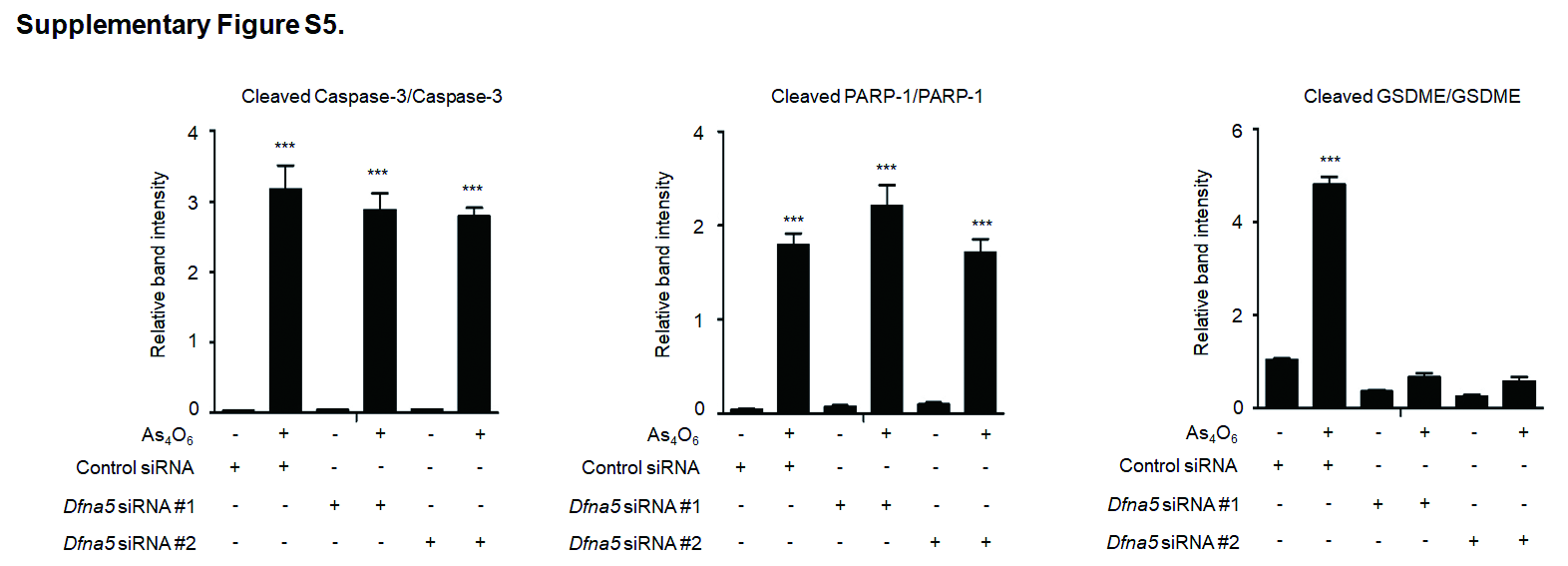

Supplement: Supplementary file 6 — Supplementary Figure S5 [file 41419_2021_3454_MOESM6_ESM.tif]

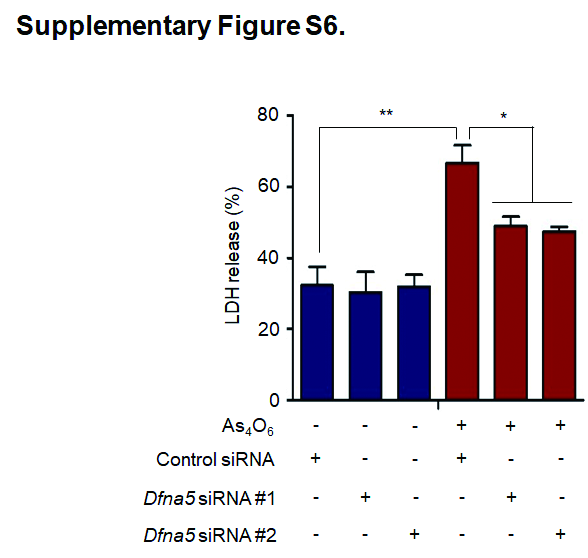

Supplement: Supplementary file 7 — Supplementary Figure S6 [file 41419_2021_3454_MOESM7_ESM.tif]

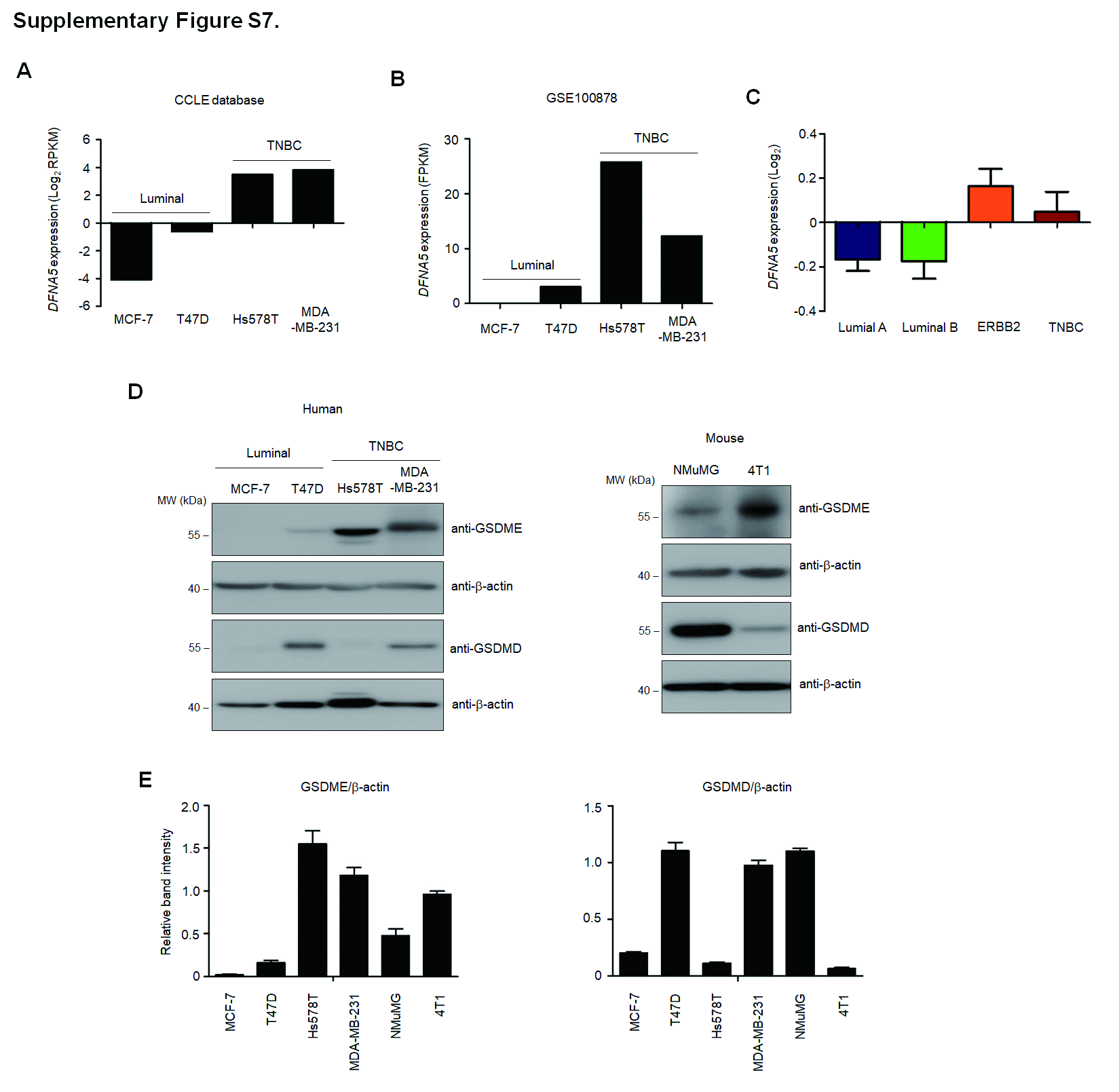

Supplement: Supplementary file 8 — Supplementary Figure S7 [file 41419_2021_3454_MOESM8_ESM.tif]

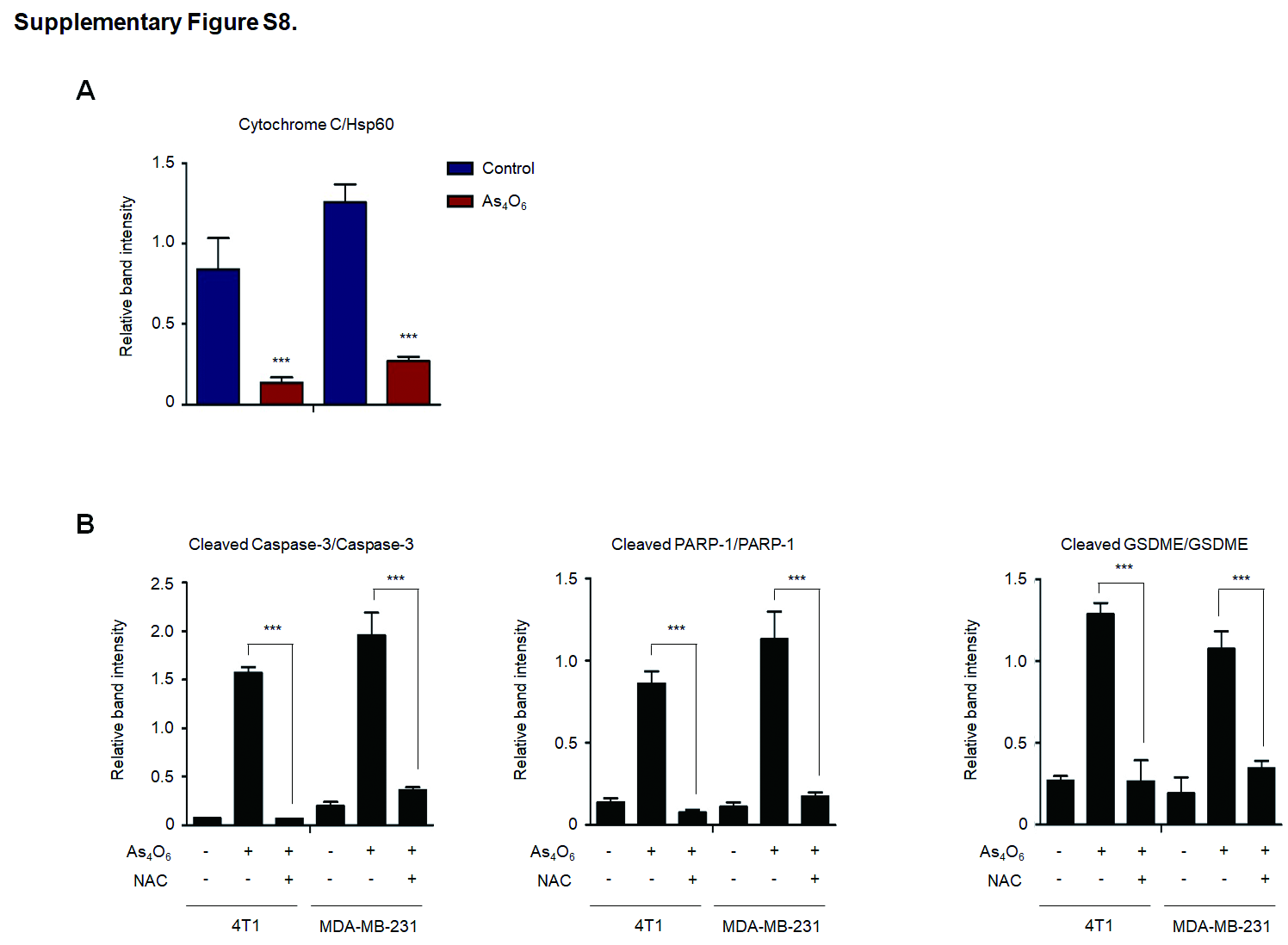

Supplement: Supplementary file 9 — Supplementary Figure S8 [file 41419_2021_3454_MOESM9_ESM.tif]

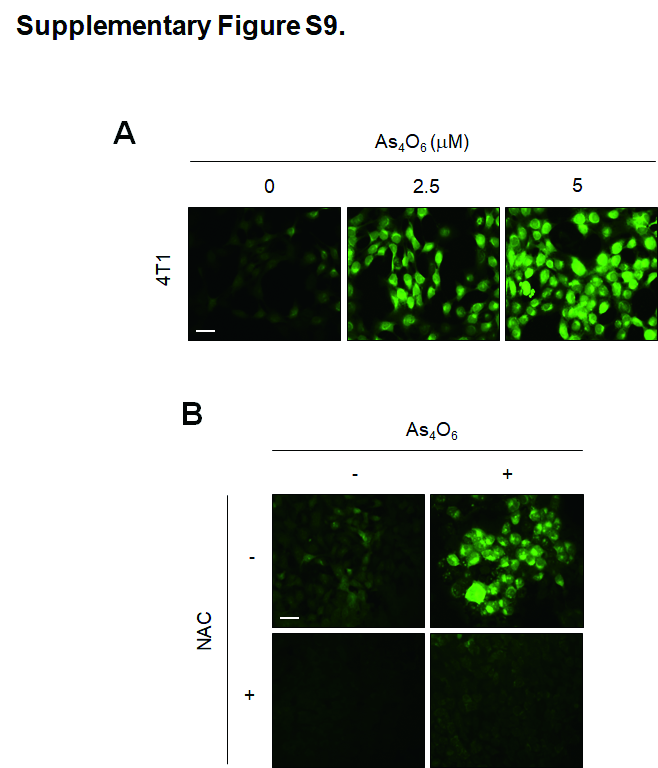

Supplement: Supplementary file 10 — Supplementary Figure S9 [file 41419_2021_3454_MOESM10_ESM.tif]

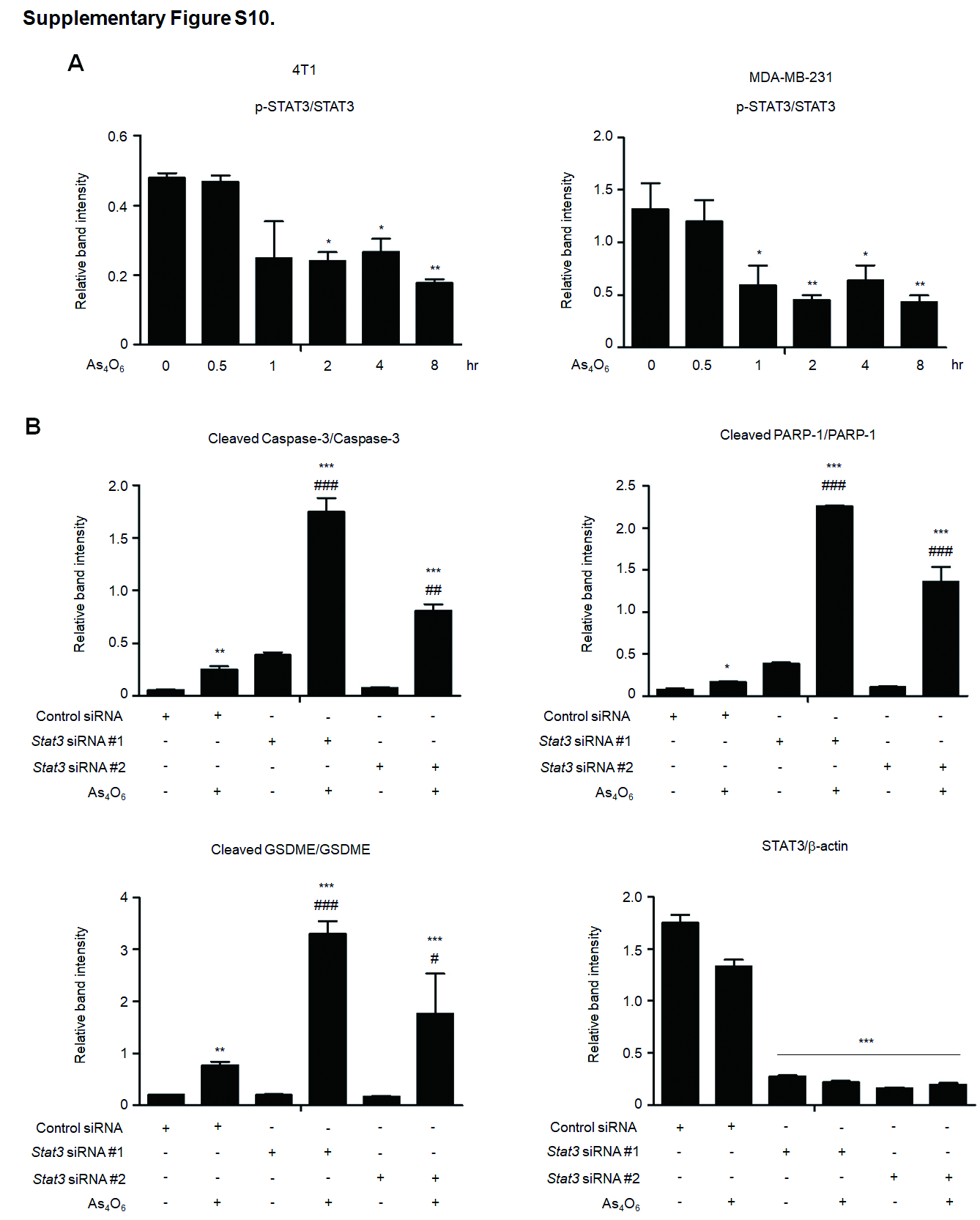

Supplement: Supplementary file 11 — Supplementary Figure S10 [file 41419_2021_3454_MOESM11_ESM.tif]

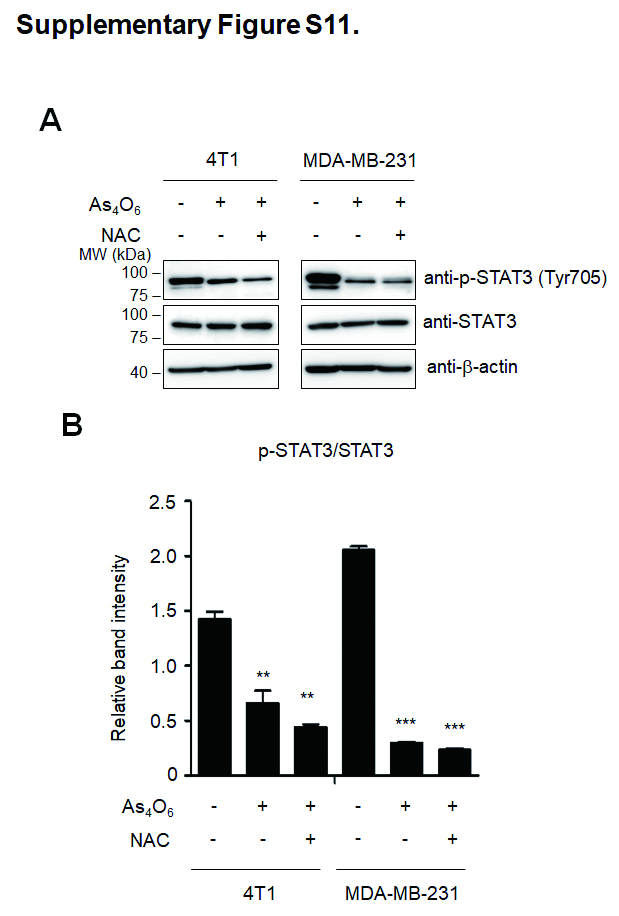

Supplement: Supplementary file 12 — Supplementary Figure S11 [file 41419_2021_3454_MOESM12_ESM.tif]

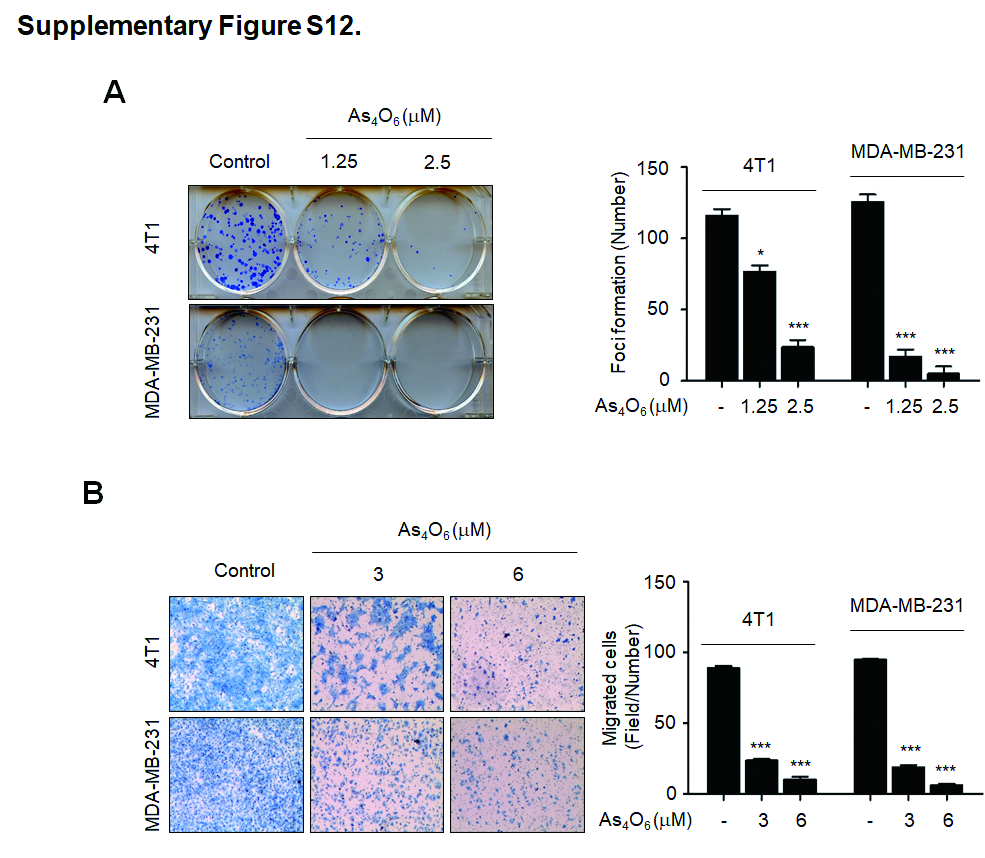

Supplement: Supplementary file 13 — Supplementary Figure S12 [file 41419_2021_3454_MOESM13_ESM.tif]
